# Near Infrared‐light responsive chlorin e6 pro‐drug micellar photodynamic therapy for oral cancer

**DOI:** 10.1002/btm2.70036

**Published:** 2025-09-16

**Authors:** Milan Paul, Swati Biswas

**Affiliations:** ^1^ Nanomedicine Research Laboratory, Department of Pharmacy Birla Institute of Technology and Science‐Pilani, Hyderabad Campus Hyderabad Telangana India

**Keywords:** chlorin e6, lung metastasis, micelles, oral cancer, photodynamic therapy

## Abstract

A major concern of conventional photodynamic therapy is its non‐specific toxicity due to off‐site drug accumulation. Micelles tend to localize the drug to the tumor site. However, rapid drug release at high concentrations from the micelles to kill the cancer cells remains a formidable task. In this manuscript, we have introduced the 2‐nitrobenzyl (2NB)‐moiety as the linker between mPEG and the photosensitizer, chlorin e6 (Ce6), to prepare the conjugate, mPEG(2‐nitrobenzyl)Ce6. We envision that 2NB as a linker between hydrophobic, Ce6, and hydrophilic mPEG would be more effective in releasing Ce6 by disassembling PEGylated 2‐nitrobenzyl chlorin e6 (mPNCe6) Ms. Characterization through Fourier transform infrared spectroscopy and ^1^H, ^13^C nuclear magnetic resonance spectra validated the successful synthesis of the conjugate. By conjugating Ce6 into the hydrophobic core of the micelles, exposure to near‐infrared light significantly hastened the dissociation of the micelles, facilitating a controlled and rapid release of Ce6's hydrophobic components within the micelles. A cellular uptake study was performed, showing that Ce6 conjugation has improved the uptake of Ce6. The cell viability assay revealed that the formulation had shown concentration‐dependent cytotoxicity upon laser irradiation. mPNCe6 group with laser irradiation has generated abundant reactive oxygen species (ROS) inside cells and exhibited green solid fluorescence, indicating the efficient delivery of Ce6 by mPNCe6 micelles and its excellent ROS generation ability inside cells upon laser irradiation. Further, in vivo studies on MOC2 tumor‐bearing mice demonstrate reduced tumor growth, lung metastasis, and drug accumulation in the tumor region. The developed nanomedicine could be a potential treatment strategy for oral cancer, minimizing the occurrence of lung metastasis.

**Translational Impact Statement**

Conventional photodynamic therapy is limited by non‐specific toxicity due to off‐target drug accumulation. This study introduces a novel nanomedicine approach using a 2‐nitrobenzyl linker between mPEG and the photosensitizer chlorin e6. This design enhances drug release upon light exposure, improving tumor localization, cellular uptake, and reactive oxygen species generation. In vivo studies demonstrated reduced tumor growth and lung metastasis in mouse oral squamous cell carcinoma cell line tumor‐bearing mice, highlighting the potential of this strategy in treating oral cancer.

**Abbreviations**
^1^H‐NMR: proton nuclear magnetic resonance
ANOVA: analysis of variance
AST: aspartate aminotransferase
BrdU: bromodeoxyuridine
BUN: blood urea nitrogen
DCC: dicyclohexylcarbodiimide
DCFH‐DA: 2′,7′‐dichlorodihydrofluorescein diacetate
EDTA: ethylenediaminetetraacetic acid
EGF: epidermal growth factor
FITC: fluorescein isothiocyanate
IC50: half maximal inhibitory concentration
ICMR: Indian Council of Medical Research
IMDM: Iscove's Modified Dulbecco's Medium
IR: infra‐red
mPEG: methoxy(polyethylene glycol)
MTT: 3‐(4,5‐dimethylthiazol‐2‐yl)‐2,5‐diphenyltetrazolium bromide
ns: not significant
OCT: optimal cutting temperature
PEG: polyethylene glycol
PLA: polylactic acid
SD: standard deviation
TUNEL: terminal deoxynucleotidyl transferase dUTP nick end labeling assay

## 1 INTRODUCTION

Cancer is the primary cause of death in affluent nations and the second most common cause of death in developing countries. Globally, around 600,000 individuals are affected by Head and Neck Squamous Cell Cancer (HNSCC), resulting in an annual mortality rate of 40%–50%.^1^ Oral cancer has a high fatality rate, especially when diagnosed at an advanced stage. Over the past few decades, the survival rates for oral cancer have only shown a slow and steady improvement despite advancements in treatment. Chemotherapy, also known as the administration of chemotherapeutic medications or antitumor agents through oral ingestion or intravenous (IV) injection, is a highly prevalent approach in the treatment of cancer and inhibiting tumor growth that has been extensively utilized in recent decades. Due to limited tumor selectivity during extended blood circulation, conventional chemotherapy medications are dispersed throughout the body, including normal tissues and organs. This leads to unwanted toxicity, severe side effects, and potential harm to the immune system.^2^ In recent years, photodynamic therapy emerged as a potential anticancer therapeutic modality due to minimal invasiveness and fewer adverse effects. A new anticancer treatment, photodynamic therapy (PDT) uses photosensitizers to convert surrounding oxygen molecules into lethal reactive oxygen species (ROS) when exposed to particular laser irradiation.^3^

Consequently, PDT, a therapy that does not require surgery, has generated significant attention for its potential to treat superficial cancers like oral squamous cell carcinoma.^4^ Due to their hydrophobic nature, tendency to aggregate easily, and insignificant payloads, photosensitizers have limited ability to accumulate and target tumors. As a result, their therapeutic effect is inadequate. However, the PDT therapy process is accompanied by continuous oxygen consumption, which exacerbates the degree of hypoxia at the tumor site, resulting in a loss in PDT efficacy. In addition, hypoxia enhances tumor metastasis and resistance to many treatments. As a result, one of the keys to improving PDT efficacy is overcoming the tumor site's hypoxia limitation.^5^ Developing appropriate drug delivery systems is necessary to address the difficulties related to inadequate solubility, limited permeability, and aggregation propensity.

Nano‐drug delivery systems are specifically engineered to enclose pharmaceuticals within their core, enhancing the drug's stability, solubility, and permeability. Furthermore, appropriately engineered nanocarriers can remain in the bloodstream for an extended period, leading to their continuous accumulation in the microenvironment of the tumor through the enhanced permeability and retention (EPR) effect. Therefore, a wide range of nanocarriers, including liposomes, polymeric micelles, nanogels, dendrimers, and polymer–drug conjugates, have been developed for delivering photosensitizers, in which the polymeric micelles were investigated extensively for drug delivery.^6^

Two primary methods have been utilized to create light‐sensitive micelles, hinging on the type of transformation induced by the photochemical event. In one approach, exposure to light prompts reversible structural shifts in photochromic groups, altering the hydrophilic–hydrophobic equilibrium and thereby transforming micellar nanostructures. These photo‐sensitive groups behave as covalent junctions between the sacrificial components and the polymer's main body. The alternative strategy involves integrating photochromic groups into the hydrophobic segment of a micelle. The photoreaction amplifies the block's polarity, disrupting the hydrophilic–hydrophobic equilibrium and rendering the liposomes block polymer chains thermodynamically unstable.^7^, ^8^, ^9^, ^10^, ^11^ The 2‐nitrobenzyl (2NB) moiety is gaining attention in drug delivery systems, particularly, its potential applications in PDT. This moiety's responsiveness to one‐ and two‐photon‐induced absorption allows for precise spatial and temporal control of drug release, which is essential in PDT. By using light‐triggered mechanisms, the 2NB linker can undergo photo‐dissociation, making it a promising candidate for targeted therapies. In PDT, light activation of photosensitizers generates ROS that induce cell death in tumor tissues.^12^ Incorporating the 2NB moiety into drug delivery systems enables the release of therapeutic agents only when irradiated, reducing off‐target effects and improving the efficacy of the treatment.^8^

Additionally, near‐infrared (NIR) light enhances tissue penetration depth, critical for treating more profound tumors in the body. The ability of the 2NB moiety to respond to both one‐photon and two‐photon absorption expands the versatility of PDT, allowing for the use of lower laser powers and minimizing damage to surrounding healthy tissues.^13^ Nevertheless, UV and visible light have primarily activated the existing light‐responsive micelles incorporating 2NB moieties, potentially causing skin damage and constraining their application in deep tissues.^14^

This study introduces a novel therapeutic approach for oral cancer treatment featuring PEGylated 2‐nitrobenzyl chlorin e6 (mPNCe6) within a single platform. This platform promises a targeted drug delivery and application of photodynamic therapy under NIR‐laser irradiation. The system exhibits prolonged blood circulation potential by utilizing mPEG as a secondary photosensitizer for chlorin e6 (Ce6). The mPNCe6 micelles present several key advantages that contribute to their therapeutic potential. Their improved water solubility ensures consistent drug dosing, while their enhanced stability prolongs drug retention, allowing for sustained and effective treatment.

Additionally, the micelles exhibit reduced systemic toxicity and improved tumor specificity, thereby minimizing unintended side effects. These micelles were subjected to detailed physicochemical characterization to thoroughly evaluate their potential, followed by in vitro and in vivo assessments using metastatic oral cancer models. The results demonstrated efficient drug accumulation in the tumor microenvironment, enhancing therapeutic efficacy while significantly reducing off‐target toxicity. This comprehensive evaluation underscores the potential of mPNCe6 micelles as a promising drug delivery system for targeted cancer therapy.

## 2 MATERIALS

### 2.1 Chemical

Vanillin, 4,6‐diamidino‐2‐phenylindole (DAPI), anhydrous *N*,*N*‐dimethylformamide (DMF), sodium borohydride, 4‐dimethylaminopyridine, paraformaldehyde, and propidium iodide (PI) were obtained from Sigma‐Aldrich, Mumbai, India. Frontier Scientific, Inc. (USA) provided Ce6. Deuterated solvents, including dimethyl sulfoxide (DMSO‐d6), deuterium oxide (D2O), and chloroform (CDCl3), were obtained from Sigma‐Aldrich (Mumbai, India). High‐purity reagents and anhydrous solvents were obtained from commercial suppliers and used as received without additional purification. 1‐Ethyl‐3‐(3‐dimethylaminopropyl)carbodiimide hydrochloride (EDC · HCl), 4‐(dimethylamino)pyridine (DMAP), trifluoroacetic acid (TFA), anhydrous dichloromethane (DCM), and DMF were procured from Sigma‐Aldrich (Mumbai, India).

### 2.2 Cell line

The mouse oral squamous cell carcinoma cell line (MOC2) was obtained from Ravindra Uppaluri's lab through Kerafast and cultured following standard protocols. MOC2 media was prepared by mixing IMDM (Iscove's Modified Dulbecco's Medium) and F12 nutrient mixture in a 2:1 ratio, supplemented with 5% fetal bovine serum (FBS) and 1% penicillin–streptomycin. The media was filtered using a 0.22 μM syringe filter before adding insulin (5 mg/mL), hydrocortisone (40 μg), and EGF (Epidermal Growth factor) (5 μg) as growth factors. Cells were maintained at 37°C in a 5% CO_2_ and 95% air environment. The FaDu cell line, derived from human hypopharyngeal carcinoma, was sourced from the American Type Culture Collection (ATCC, USA; Cat. No. HTB‐43). Dulbecco's Modified Eagle's Medium (DMEM; Cat. No. AL007A), Minimum Essential Medium Eagle (MEM; Cat. No. AT219), penicillin–streptomycin (Cat. No. A001), FBS (Cat. No. RM10432), and trypsin–EDTA (Ethylenediaminetetraacetic acid) (Cat. No. TCL007) were purchased from Himedia Labs (India). The cells were cultured under standard conditions, maintaining 37°C, 5% CO_2_, and 90% humidity in a controlled incubator. Dialysis membranes were purchased from Spectrum Laboratories Inc. (Rancho Dominguez, CA, USA).

### 2.3 Animal

Female C57BL/6 mice, aged 4–6 weeks, were procured from the ICMR National Animal Resource Facility for Biomedical Research (NARFBR), Hyderabad, to evaluate the efficacy of antitumor therapies. All pharmacological studies were approved by the Institutional Animal Ethics Committee at BITS‐Pilani, Hyderabad (Approval No. BITS‐Hyd/IAEC/2023/36) and adhered to ethical guidelines. The mice were housed in standard cages, five per cage, under controlled conditions of 23–24°C with 50%–60% relative humidity, maintained on a 12‐h light/dark cycle with ad libitum access to food and water.

## 3 METHODS

### 3.1 Synthesis of mPNCe6 and mPCe6 conjugate

#### 3.1.1 Synthesis of ethyl 2‐(4‐formyl‐2‐methoxyphenyl) acetate

**SCHEME 1:**
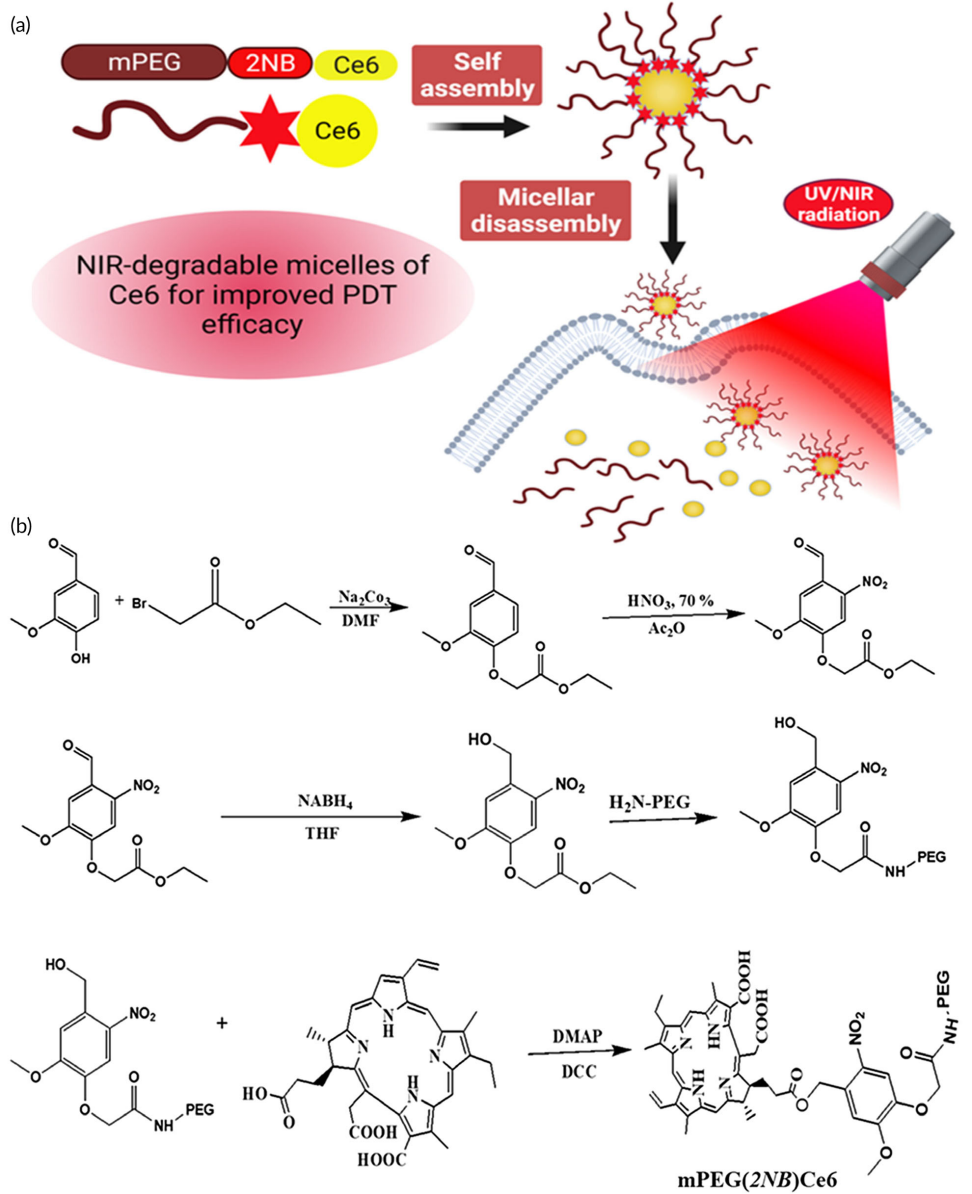
Preparation of PEGylated 2‐nitrobenzyl chlorin e6 (mPNCe6) micelles (a); synthesis of methoxy(polyethylene glycol)‐*2‐nitrobenzyl*‐chlorin e6 (mPEG‐2NB‐Ce6) conjugate (b). 2NB, 2‐nitrobenzyl; Ce6, chlorin e6; DMAP, 4‐(dimethylamino)pyridine; DMF, *N*,*N*‐dimethylformamide, NIR, near‐infrared; PDT, photodynamic therapy.

The Scheme 1 is as follows to prepare the NIR‐light cleavable amphiphilic polymer. A mixture of sodium carbonate (Na_2_CO_3_) (3.5 g, 33 mmol), vanillin (2.5 g, 16 mmol), and ethyl bromoacetate (2 mL, 3 g, 18 mmol) was dissolved using dimethylformamide (DMF) (15 mL) as a solvent and stirred at room temperature (RT) for 24 h. The above mixture was partitioned using ethyl acetate (EtOAc) and saturated sodium chloride solution. The formed aldehyde ester was dried and the organic phase was removed with anhydrous sodium sulfate. The yield was observed to be 90.5%. ^1^H‐NMR, IR was used to characterize the product.

#### 3.1.2 Synthesis of 2‐(4‐formyl‐2‐methoxy‐5‐nitrophenoxy)acetic acid

A mixture was prepared using acetic anhydride (10 mL) and aldehyde ester 1 (3.5 g, 15 mmol). Cool 70% HNO_3_ (10 mL) and acetic anhydride (15 mL) were added to the solution, maintaining the temperature at 4°C. After 2 h of stirring, the above mixture was allowed to warm up to RT. After the temperature was set, stir the mix for an additional 4 h and then add cold water to it. The pH was adjusted to 13–14 using sodium hydroxide and then changed to pH 2–3 using 36% HCl. The aqueous phase was extracted with EtOAc, and the organic phase was dried and evaporated to yield a yellow solid. To ensure the purity of the product, recrystallization was performed using methanol/water (MeOH/H_2_O) to afford the pure nitro‐acid 2 (3 g, 12 mmol, 80% yield). ^1^H‐NMR, IR characterized the product.

#### 3.1.3 Synthesis of 2‐(4‐(hydroxymethyl)‐2‐methoxy‐5‐nitrophenoxy)acetic acid

Into the solution of 2‐(4‐formyl‐2‐methoxy‐5‐nitrophenoxy)acetic acid (1 g) in THF (20 mL), sodium borohydride (NaBH_4_) was added at RT and allowed to stir for 24 h. Next, 1 M HCl was added to adjust the pH in the range of 2–3. The organic phase was dried and evaporated, and the aqueous phase was extracted using EtOAc. A pale‐yellow solid was obtained with a 95% yield. ^1^H‐NMR IR characterized the product.

#### 3.1.4 Synthesis of PEG‐NH_2_ ‐(4‐(hydroxymethyl)‐2‐methoxy‐5‐nitrophenoxy)acetic acid

PEG‐NH_2_ (0.4 g, 0.54 mmol), triethylamine (0.075 mL, 0.055 g, 0.54 mmol), EDC (0.1 g, 0.52 mmol), and 2‐(4‐(hydroxymethyl)‐2‐methoxy‐5‐nitrophenoxy)acetic acid were mixed with DMF at RT and stirred for 24 h. The mixture was then partitioned between the saturated sodium chloride solution and EtOAc. The organic phase was dried and evaporated for the product (71% yield). ^1^H‐NMR and IR characterized the product.

#### 3.1.5 Synthesis of methoxy(polyethylene glycol)‐*2‐nitrobenzyl*‐chlorin e6

Ce6 (20 mg) was dissolved into the solution of DCC (dicyclohexylcarbodiimide) and DMAP in DMF to activate the carboxylic group of Ce6. After 3 h, PEG‐NH2‐(4‐(hydroxymethyl)‐2‐methoxy‐5‐nitrophenoxy)acetic acid was slowly added. The reaction mixture was kept under stirring for 24 h at RT. DMF was evaporated using a rotary evaporator. Further, Milli‐Q water was added and dialyzed using a cellulose ester membrane (24 h, 1 KDa). A solid powder was obtained after lyophilization with a 79% yield. ^1^H‐NMR, IR characterized the product.

#### 3.1.6 Synthesis of mPEG‐PLA

As previously outlined in the literature,^15^ we employed a ring‐opening polymerization approach for the synthesis of the mPEG‐PLA copolymer. In brief, 500 mg of mPEG and 200 mg of D,L‐lactide were combined in a polymerization tube, and 0.004% w/w of stannous octoate served as a catalyst. The mixture underwent stirring for 6 h at 160°C. The product obtained was reconstituted and precipitated with tetrahydrofuran and ice‐cold diethyl ether, respectively. Finally, dialysis against water was done for 24 h using a dialysis bag (molecular weight cutoff [MWCO] 12–14 KDa, Spectrum Laboratories, Inc.) made up of a cellulose ester membrane. The dialysis was performed as a part of the reaction to remove any unreacted starting materials. After dialysis, the contents were dried using vacuum drying. The formed polymer solution was then dissolved in Milli‐Q water and kept for lyophilization, yielding a white, fluffy polymer. ^1^H‐NMR and IR characterized the product.

#### 3.1.7 Synthesis of mPEG‐PLA‐Ce6

The hydroxyl group of mPEG‐PLA (150 mg) dissolved in dimethylformamide (2 mL), along with DMAP (24.43 mg), underwent conjugation with a total of 29.83 mg of Ce6, pre‐activated with 38.27 mg of EDC in 3 mL of dimethylformamide, for 1 h in the dark at RT. Subsequently, the reaction was carried out in the dark at RT for 12 h. Then, by the dialysis using a dialysis bag of MWCO of 12,000–14,000 Da, the remaining unreacted Ce6 was removed. To eliminate any remaining unreacted Ce6 under sink conditions, the product underwent extensive dialysis for 72 h with regular water changes. The final step involved lyophilization to obtain mPEG‐PLA‐Ce6. ^1^H‐NMR and IR characterized the product.

### 3.2 Preparation of methoxy(polyethylene glycol)‐*2‐nitrobenzyl*‐chlorin e6 (mPNCe6 micelles) and mPEG‐Ce6 (mPCe6) micelles

The methoxy(polyethylene glycol)‐*2‐nitrobenzyl*‐chlorin e6 (mPEG‐2NB‐Ce6) (mPNCe6) conjugate and mPCe6 conjugate were dissolved in DMF (10.0 mg/mL), and by a rotary evaporator, the solvent was removed. The film of polymer formed was hydrated with 500 μL phosphate‐buffered saline (PBS) (pH 7.4) by stirring at 37°C for 5 h. At the end, the unencapsulated Ce6 was removed from the micellar solution of mPNCe6 and mPCe6 by centrifugation at 13,000 RPM at 4°C for 20 min and filtration through a 0.45 μm filter.

#### 3.2.1 Characterization of micelles

##### Physicochemical characterization

The size distribution (by number) and zeta potential of mPCe6 and mPNCe6 micelles were analyzed using the Zetasizer Nano ZS (Malvern Instruments, UK), equipped with a 50 mW He‐Ne laser (633 nm). For size measurements, micelles were dissolved in deionized water (18.2 MΩ) at a final concentration of 0.2 mg/mL, while zeta potential measurements were performed in 10 mM NaCl at the same concentration. Both samples were filtered using 0.2 μm syringe filters, and measurements were conducted in Malvern Zetasizer Nano series disposable folded capillary cells. Each measurement was performed in triplicate to ensure accuracy.

Drug loading (DL) and entrapment efficiency play a crucial role in the characterization of micelle and they also define the performance of that formulation. For the mPNCe6 and mPCe6 Ms, the DL and entrapment efficiency were determined at 405 nm wavelength by using a UV‐Vis spectrophotometer (Shimadzu, Germany). The λ_max_ for the free drug was defined by the standard calibration curve in DMSO. The amount of the drug entrapped in carrier system and present in supernatant helps in the calculation of %DL and entrapment efficiency % using previously reported formulae.

The critical micelle concentration (CMC) of the mPNCe6 and mPCe6 Ms was determined using pyrene as the hydrophobic probe and a microplate reader (SpectramaxTM, Molecular Devices, USA).^16^ The excitation wavelength of the solution was set to 339 nm and a slit width of 5 nm for both excitation and emission. Pyrene solution (50 L; 10 mg/mL in CDCl3) was combined with a micellar solution at concentrations ranging from 3.125 to 100 mg/mL. The graph was plotted between log concentrations and fluorescence intensities of mPNCe6, mPCe6 Ms, at ratios of I338/I390 at the *x* and *y* axes, respectively. The surface tension was determined by identifying functional groups and examining their interactions for Ce6, mPNCe6, and mPCe6 were carried out using Fourier transform infrared spectroscopy (FTIR) using the KBr pellet technique. The analysis utilized an FTIR spectrometer (Jasco‐4200, USA). The FTIR grade KBr was blended with the freeze‐dried samples and subjected to scanning across the 4000–400 cm^−1^ frequency range at a resolution of 4 cm^−1^ (64 scans) to generate spectra.^17^ The surface morphology of mPNCe6 and mPCe6 micelles was investigated using Field Emission Scanning Electron Microscopy (FESEM) following irradiation with a 660 nm laser at a power density of 0.5 W/cm^2^ for 5 min. To prepare the samples, a thin layer of micelles was evenly spread over adhesive carbon tape mounted on aluminum stubs. The analysis was performed in accordance with established protocols.^18^

##### In vitro drug release studies

The in vitro release of Ce6 from mPNCe6 and mPCe6 micelles was assessed using the dialysis bag method at 37°C in PBS with pH values of 7.4, 6.5, and 5.5.^19^ Dialysis bags with a MWCO of 3.5 kDa were loaded with different ratios of mPNCe6/mPCe6 micelles and free Ce6 at a concentration of 50 μg/mL. Laser irradiation was applied for 5 min using a 660 nm laser at a power density of 0.5 W/cm^2^. The bags were then submerged in PBS at the respective pH conditions, stirred continuously at 300 RPM, and incubated at 37°C. At predetermined time points, 1 mL of the release medium was collected and replaced with fresh PBS. The amount of released Ce6 was quantified using a UV–visible spectrophotometer at a wavelength of 405 nm.

##### Hemolysis assay

The hemolysis assay was performed to evaluate the potential toxicity of the micelles on red blood cells (RBCs). The 5 mL rat blood was initially collected in EDTA‐containing tubes. Then, the RBCs were isolated by centrifugation at 3000 rpm at 4°C for 30 min.^20^ After thorough washing, the pellet was resuspended in PBS (5% v/v suspension). Then the 5% v/v RBC solution was incubated with mPCe6 Ms, mPNCe6 Ms at different concentrations for 2 h at RT. Saline was used as a negative control, and 1% w/v Triton‐X 100 was used as a positive control. The hemoglobin (Hb) content in the supernatant was quantified using a Spectramax microplate reader (Molecular Devices, USA). The percentage of hemolysis induced by the formulation was calculated using the following equation:

$$\text{Hemolysis}\;\left(\%\right)=\frac{\mathrm{Ab}\;\text{sample}-\mathrm{Ab}\;\left(-\right)\;\text{control}}{(\mathrm{Ab}\;\left(+\right)\;\text{control}-\mathrm{Ab}\;\left(-\right)\;\text{control)}}\times 100$$

where: Ab sample: absorbance of the sample. Ab (−) control: absorbance of negative control. Ab (+) control: absorbance of positive control.

### 3.3 Biochemical characterization

#### 3.3.1 Findings of singlet oxygen generation

Singlet oxygen (^1^O_2_) generation (SOG) was assessed using three distinct probes: *p*‐nitrosodimethylaniline (RNO), 9,10‐dimethylanthracene (DMA), and Singlet Oxygen Sensor Green (SOSG), each utilizing different mechanisms to confirm (^1^O_2_) production upon micelle activation. For the RNO assay, RNO undergoes bleaching in the presence of ^1^O_2_, leading to a measurable decrease in absorbance. A reaction mixture containing RNO (20 μM), imidazole (50 μM), and mPCe6/mPNCe6 micelles (Ce6 equivalent of 6 μg/mL) was prepared in PBS (pH 7.4). The samples were exposed to a 660 nm laser (0.5 W/cm^2^) for a defined duration, and the absorbance at 440 nm was recorded at regular intervals using a UV–visible spectrophotometer. The reduction in RNO absorbance confirmed ^1^O_2_‐mediated oxidation.^21^

For the DMA assay, DMA serves as a selective (^1^O_2_) scavenger, undergoing oxidative degradation to form endoperoxides, which results in a decline in its absorbance at 378 nm. A mixture of DMA (50 μM) and micelles (Ce6 @ 6 μg/mL) in PBS was irradiated using a 660 nm laser, and the absorbance was measured over time. The rate of decrease in absorbance indicated the extent of (^1^O_2_) generation.

For the SOSG assay, the fluorescence‐based detection of ^1^O_2_ was carried out using SOSG (2.5 μM) mixed with mPCe6/mPNCe6 micelles (Ce6 @ 6 μg/mL) in deionized water. Upon 660 nm laser exposure, (^1^O_2_) reacts with SOSG, forming a fluorescent endoperoxide (SOSG‐EP). The fluorescence intensity of SOSG‐EP (Ex: 510 nm, Em: 525–536 nm) was measured using a spectrofluorometer. The increase in fluorescence intensity directly correlated with the amount of (^1^O_2_) generated. The ^1^O_2_ generation mechanism involves the excitation of Ce6 molecules within micelles upon 660 nm laser irradiation, transitioning them from the ground state (^1^S_0_) to an excited singlet state (^1^S_1_). Through intersystem crossing, Ce6 undergoes a transition to the triplet state (^3^T_1_), which then interacts with molecular oxygen (O_2_), converting it into highly reactive ^1^O_2_. This (^1^O_2_) interacts with probes such as RNO, DMA, and SOSG, leading to measurable fluorescence changes, validating the photodynamic activity of mPCe6 and mPNCe6 micelles.

### 3.4 In vitro tissue culture study

#### 3.4.1 Fluorescence imaging and flow cytometry of mPNCe6 and mPCe6 micelles uptake

FaDu and MOC2 cells (50,000 cells/well) were cultured on coverslips within 12‐well plates. Cells were treated with Ce6, mPNCe6, and mPCe6 micelles (6 μg/mL Ce6) for durations ranging from 1 to 4 h. The cells were washed with PBS, DAPI staining, and 4% paraformaldehyde fixation. Using Fluoromount G, the cells were put on a glass slide so they could be seen under a fluorescence microscope (Leica, Germany). For flow cytometry studies, cells were seeded at 0.5 × 10^6^ cells/well density in six‐well plates. Cells were handled in the same manner as the fluorescence microscopy investigation the next day. At the final treatment stage, cells were washed with PBS, trypsinizing, centrifugation, rinsing, and PBS (pH 7.4) resuspending. Using a flow cytometer (BD FACS Aria III), the fluorescence of 10,000 viable cells was measured to assess cellular uptake. D FACS Diva software was used to process the data.

#### 3.4.2 In vitro cytotoxicity study

An in vitro phototoxicity experiment with free Ce6, mPCe6, and mPNCe6 Ms was carried out on FaDu and MOC2 cells.^22^ Ten thousand cells per well were plated in 96‐well plates for cell adhesion and kept at 37°C overnight. After rinsing the cells in PBS, each well was filled with 100 μL of free Ce6, mPCe6, and mPNCe6 Ms, which were incubated for 12 h. Following the incubation, the cells were exposed to a 660 nm laser at a 0.5 W/cm^2^ power for 2 min. Irradiated cells were then incubated in the dark for an additional 12 h at 37°C. After that, the culture medium was withdrawn, and 5 mg/mL, 50 μL MTT (3‐(4,5‐dimethylthiazol‐2‐yl)‐2,5‐diphenyltetrazolium bromide) was added to each well post 3 h incubation to dissolve purple formazan crystals formed by MTT reduction by live cells' mitochondria; 150 μL of DMSO was supplied to each well. Absorbance measurements were taken at 570 nm to assess the sample, and background absorbance was recorded at 630 nm. The background value was subtracted from the 570 nm reading to account for any non‐specific absorbance. Cell viability was then calculated using a standard formula, as previously reported, which compares the absorbance of treated samples to that of control samples, providing a reliable measure of cell health and viability.

#### 3.4.3 ROS findings

In six‐well plates, cells of the FaDu and MOC2 types were seeded at a density of 1 × 10^4^ cells/well using the complete media.^23^ The cells were treated with free Ce6, mPCe6, and mPNCe6 Ms the next day, with a Ce6 concentration of 50 μg/mL for a full day. After that, the cells were exposed to radiation for 5 min at a power density of 0.5 W/cm^2^ using a 660 nm laser or not. Following that, confocal images were collected before and after irradiation. The changes in fluorescence intensity were also recorded at the beginning and finish of the photoreaction.

#### 3.4.4 Mitochondrial membrane potential study

For this experiment, FaDu and MOC2 cells were seeded (1 × 10^5^ cells/well) in complete media on six‐well assay plates. Treatment of free Ce6, mPCe6, and mPNCe6 micelles at a Ce6 concentration of 10 μM was given to the cells and incubated for 24 h at 37°C. Then, they were rinsed in cold PBS and stained for 30 min in a CO_2_ incubator with 2 M of 5,5′,6,6′‐tetrachloro‐1,1′,3,3′tetraethyl benzimidazole carbocyanine iodide (JC1 dye). The cells were then examined under fluorescence microscopy with 530 for green and 630 for red. After processing the pictures using Image J software, the yellow signal represented green and red co‐localization.

#### 3.4.5 Apoptosis study

FaDu and MOC2 cells were seeded in 12‐well plates and allowed to grow overnight to ensure proper attachment.^24^ The cells were then treated with either free Ce6, mPCe6, or mPNCe6 micelles, each containing Ce6 at a concentration of 6 μg/mL with or without laser exposure at 660 nm and 0.5 W/cm^2^ for 5 min. After 18 h of incubation, the treatment medium was removed, and the cells were carefully washed with PBS (pH 7.4) to eliminate any unbound or residual treatment. Following the washing steps, the cells were collected by centrifugation to proceed with further analysis. The pellet formed was resuspended in 100 μL of binding buffer along with 1 μL of Annexin V FITC (Fluorescein isothiocyanate) and 10 μL of PI, as described in the TACS® Annexin V‐FITC Apoptosis Detection Kit. The cells were analyzed using flow cytometry (Flowsight Amnis, Millipore, USA) by gating 10,000 live cells after 15 min of incubation.

#### 3.4.6 Cell cycle findings

Free Ce6, mPCe6, and mPNCe6 Ms were incubated overnight with 0.5 × 10^6^ cells/well at a Ce6 concentration of 6 μg/mL. The cells were collected by trypsin, and subsequently, the cell pellet was subjected to washing with ice‐cold PBS. Then they were fixed in 70% ethanol with gentle agitation. Overnight, the samples were maintained at 20°C. The next day, the centrifugation was carried out for fixed cells at 1000 rpm, 4°C for 7 min. Ultimately, the cells were resuspended in staining solution (500 μL) containing 20% w/v RNAase, 0.1% v/v Triton X‐100, and 2% w/v PI in PBS (pH 7.4). The incubation of samples was performed at RT for 30 min in the dark before being analyzed using flow cytometry (BDAriaTMIII). A dot plot of the PI width against the PI area was made. A histogram of the PI area was shown on the *x*‐axis, with counts on the *y*‐axis.

#### 3.4.7 Three‐dimensional sphere assay

For the three‐dimensional sphere assay, the seeding of cells in 50% precoated Matrigel (Corning) and 50% of medium without serum was executed initially.^25^ For every 3 days, the 5% FBS was added as the culture medium supplement and 2% Matrigel was replaced along with it. The treatment has been started after 48 h of seeding in the plate. Finally, using the inverted phase contrast microscope, Leica, Germany, the three‐dimensional (3D) culture experiments were imaged and scored based on the integrity of the 3D structure. Around 50 structures were scored for each drug treatment.

### 3.5 Syngeneic tumor model using MOC2 cells

#### 3.5.1 In vivo micelles biodistribution and efficacy studies

The therapeutic efficacy of the developed formulations was evaluated in vivo using a male C57BL/6 mouse model. The lower part of the mice's body was shaved before subcutaneous injection. The growth of MOC2 cells was maintained as mentioned above. About 2 × 10^6^ cells suspension/100 μL of sterile PBS was injected subcutaneously into the lower flank of the animals.^26^ During the procedure, mice were anesthetized with inhaled 5% isoflurane. To determine tumor volume, the greatest longitudinal diameter (length) and the greatest transverse diameter (width) were measured with an external caliper. Tumor volumes were based on caliper measurements and were calculated using the following formula: tumor volume = length × width^2^ × 0.5. The mice were then divided into four groups, each consisting of five mice: control (receiving PBS), free Ce6 (+L), mPCe6 Ms (+L), and mPNCe6 Ms (+L), with a Ce6 dose of 5 mg/kg. Treatment was administered intravenously every other day for 21 days. After each formulation administration, the tumor site was exposed to a 660 nm laser (0.5 W/cm^2^) for 5 min. Tumor size was measured every other day, and body weight was monitored throughout the study. After 21 days, the mice were euthanized, and their tumors, along with major organs (liver, heart, spleen, kidney, and lungs), were collected for further imaging and analysis. The enumeration of macroscopic metastatic nodules in lung tissue served as a metric to assess the inhibitory effects of nanoparticle compositions on the incidence of oral cancer lung metastases. The in vivo tumor accumulation of free Ce6, mPCe6, and mPNCe6 micelles was also identified. Biodistribution analysis of free Ce6, mPCe6, and mPNCe6 Ms was performed in mice over a time period of 36 h. The fluorescence emitted by Ce6 was captured at 1, 3, 6, 12, 24, and 36 h post‐administration.^27^ Post imaging, the mice were euthanized, and the tumors and primary organs (liver, heart, spleen, kidney, and lung) were collected for further studies.

#### 3.5.2 Immunohistochemistry findings

The excised tumors were preserved in OCT (Optimal Cutting Temperature) medium and cryo‐sectioned into 5 μm thick slices using a Leica CM1950 cryotome (Leica Biosystems, Germany). To evaluate the therapeutic efficacy of mPCe6 and mPNCe6 micelles, apoptosis and proliferation markers were assessed using a TUNEL (Terminal deoxynucleotidyl transferase dUTP nick end labeling) kit and KI‐67 immunostaining, respectively.^28^ For KI‐67 analysis, tumor sections were first coated with a blocking buffer to prevent non‐specific binding. The slices were then incubated overnight at 4°C with the KI‐67 primary antibody (Rabbit mAb #9129S). Following three PBS washes, a fluorescently labeled secondary antibody (Alexa Fluor Plus 488) was applied for 2 h at 25°C while shielding the samples from light. After incubation, the sections were washed again with PBS and counterstained with DAPI for 5 min before imaging under a fluorescence microscope. The TUNEL assay was conducted according to the protocol of the Apoptosis Detection Kit (Merck, Darmstadt, Germany). Tumor sections were incubated with the terminal deoxynucleotidyl transferase (TdT) enzyme at 37°C for 1 h. The TUNEL reaction mixture was then applied, and the slides were kept in a dark, humid chamber at 37°C for another hour. After three PBS washes, the sections were stained with DAPI for 5 min and visualized under a fluorescence microscope. For ROS detection, mice with tumors of approximately 300 mm^3^ were treated with free Ce6 (+L), mPCe6 Ms (+L), and mPNCe6 Ms (+L), receiving a Ce6 dose of 5 mg/kg followed by laser irradiation (660 nm, 0.5 W/cm^2^, for 5 min). At 24 h post‐injection, a 25 μM DCFH‐DA (2',7'‐dichlorodihydrofluorescein diacetate) solution (50 μL) was directly injected into the tumor. After 30 min, tumors were excised, embedded in OCT medium, and sectioned into 5 μm slices using a cryotome. The sections were then examined under a fluorescence microscope, primarily using the FITC channel (Ex/Em: 495/519 nm). Fluorescence intensities were quantified and analyzed using ImageJ software.

##### Efficacy and safety in MOC2 mouse model

To determine the systemic effect of intravenously administered drugs and formulations in tumor‐bearing mice, the investigators can use serum and hematological assessments. The drugs and formulations, such as free Ce6, mPCe6, and mPNCe6 Ms, were assessed for their systemic effect. The plasma was isolated from the blood sample (0.5 mL) collected at specific intervals through the retro‐orbital plexus with heparinized tubes. The corresponding diagnostic kit quantified the creatinine and BUN, and the kidney tissues were examined macroscopically to observe the changes.

### 3.6 Statistical analysis

Experiments were performed in triplicates. All the data were expressed as mean values with standard deviation (mean ± SD). To determine the significance, one‐way ANOVA and Student *t*‐tests were conducted between the groups using GraphPad Prism (GraphPad, Inc., California, USA). The probability value (*p*‐value) of <0.05, <0.01, and <0.001 represents statistically significant. *, **, and ***.

## 4 RESULTS AND DISCUSSION

### 4.1 Characterization of mPNCe6 and mPCe6 conjugate

We have introduced the 2NB‐moiety as the linker between mPEG and the photosensitizer, Ce6, to prepare the conjugate, mPEG‐2NB‐Ce6. We envision that 2NB as a linker between hydrophobic Ce6 and hydrophilic mPEG would be more effective in releasing Ce6 by disassembly than mPEG‐PLA‐2NB. The mPEG‐2NB‐Ce6 conjugate was synthesized using the procedure illustrated in Scheme 1. Photo‐dissociation to the 2NB linker was induced by NIR irradiation of N2 at low power via a multi‐photonic excitation process, followed by the detachment of bulky PEG derivatives from the surfaces of the Ms and resulting in the Ce6 release from the Ms‐core. This synthetic route encompassed several critical steps in the development of nano Ms. The structural characterization of the synthesized micelles was performed using ^1^H‐NMR and FTIR spectroscopy to confirm the successful incorporation of PEG and Ce6. In the ^1^H‐NMR spectrum, the ^1^H‐NMR showed that the presence of PEG protons was observed within the 2.5–3.5 ppm range, attributed to the methylene (‐CH_2_‐) proton signals. The presence of Ce6 in the mPN copolymer was verified by the appearance of two distinct peaks at 5.2 and 6.3 ppm, which correspond to the methane (‐CH‐) protons of the Ce6 structure (Figure S1F). Further validation was obtained through FTIR spectroscopy, which provided insights into the functional groups involved in the polymer conjugation. The peaks at 2988 and 1720 cm^−1^ were attributed to the C–H stretching vibrations and C=O stretching of PEG‐NH_2_, respectively. Additionally, bands observed at 1079 and 1248 cm^−1^ were assigned to the C–O–C stretching of PEG and PLA, confirming their integration into the mPNCe6 polymeric structure (Figure S2E). Notably, the presence of distinct peaks at 1722 and 1248 cm^−1^, characteristic of the ester bond (O=C–O), further substantiated the successful conjugation of mPEG and Ce6 within the micelle formulation.

### 4.2 Physiochemical characterization of micelles

**FIGURE 1:**
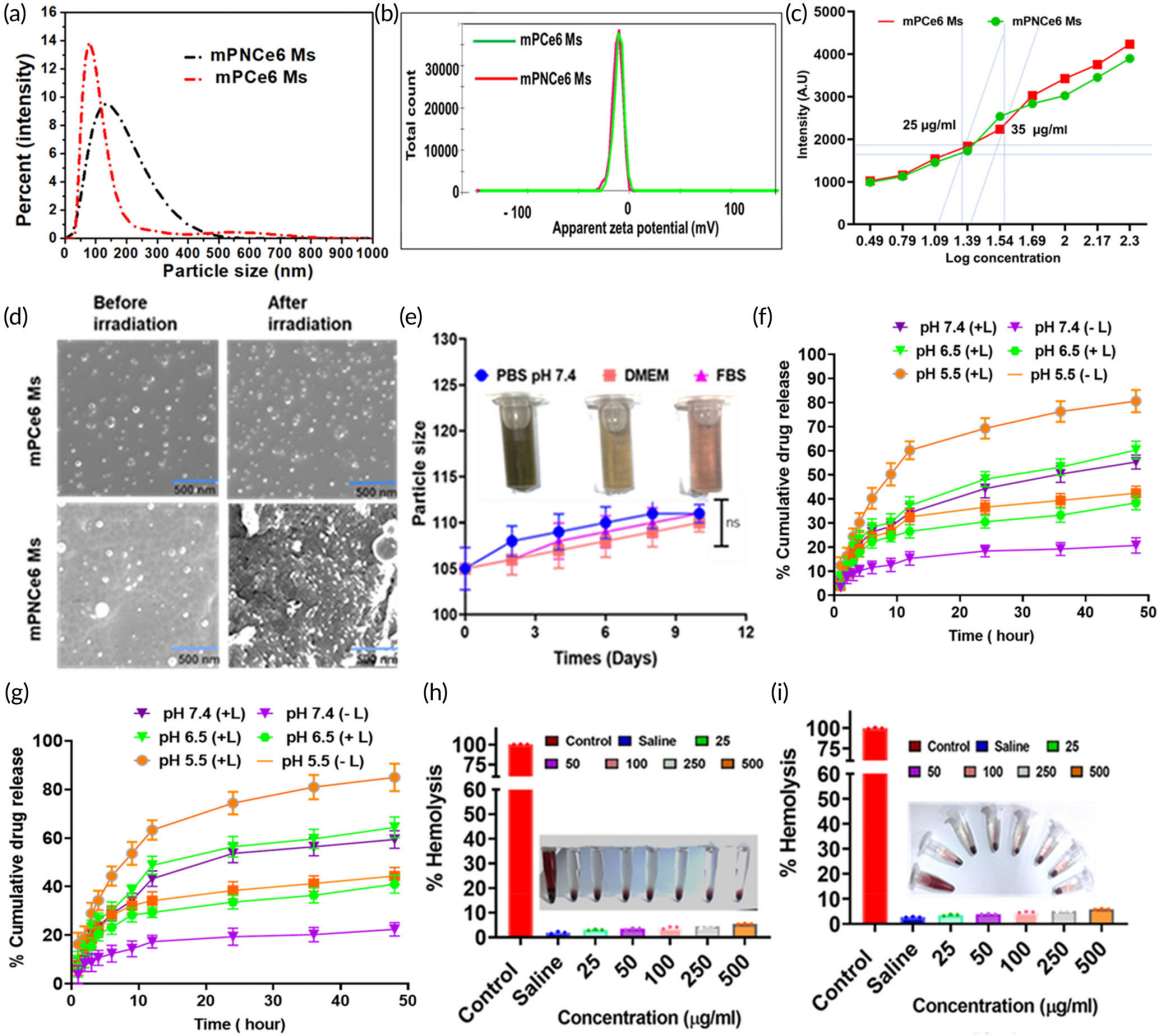
(a) Particle size analysis comparing mPCe6 Ms and PEGylated 2‐nitrobenzyl chlorin e6 (mPNCe6) Ms, illustrating the differences in size distribution between the two formulations. (b) Zeta potential measurements of mPCe6 Ms and mPNCe6 Ms, indicating the surface charge and stability of the micelles. (c) The critical micelles concentration analysis of mPCe6 Ms and mPNCe6 Ms. (d) Scanning electron microscopy images of mPCe6 Ms and mPNCe6 Ms. (e) Stability study of mPNCe6 Ms over time, demonstrating the physical stability of the formulation under different conditions. (f and g) In vitro *drug release* profile of mPCe6 Ms and mPNCe6 Ms with or without irradiation. (h and i) Hemolysis assay results for mPCe6 Ms and mPNCe6 Ms, evaluating the biocompatibility and safety of the micelles in terms of red blood cell lysis. FBS, fetal bovine serum; DMEM, Dulbecco's Modified Eagle's Medium; PBS, phosphate‐buffered saline.

The particle size of mPCe6 and mPNCe6 Ms was 111.8 ± 3.24 and 138.43 ± 2.45, respectively, as measured by the dynamic light scattering technique. (Figure 1a) The zeta potential was −13.4 ± 3.65 mV for mPCe6, and −15.6 ± 1.74 mV for mPNCe6 Ms (Figure 1b). The morphology of the prepared micelle was found to be spherical. The encapsulation efficiency (EE) and %DL of mPCe6 and mPNCe6 micelles were determined to be 76.35% ± 1.85% and 84.63% ± 3.74%, respectively, while the DL capacities were measured at 7.6% ± 1.05% for mPCe6 and 8.6% ± 0.95% for mPNCe6. Figure 1c represents the determination of the CMC using the surface tension and pyrene method. The surface tension of a series of increasing concentrations of polymers was measured. As shown in Figure S3, before CMC, the surface tension decreased with increasing concentration, and after CMC, the surface tension became constant. mPCe6 and mPNCe6 exhibited CMC values of 25 and 35 μg/mL. The average particle size of mPNCe6 Ms was 110 nm, and their shape was spheroidal. The best nanomaterial size for obtaining the EPR effect has been shown to be between 10 and 200 nm, indicating that mPNCe6 Ms can target tumor tissues passively. The stability study was conducted over 7 days, and the particle size showed no significant increase in PBS, FBS, and DMEM media (Figure 1e). No significant increase in particle size was observed in any of the media over the 7‐day period, demonstrating the formulation's stability. In assessing mPCe6 Ms' reaction to NIR activation, scanning electron microscopy (SEM) was utilized to analyze the microstructures of the polymeric micelles (Figure 1d). The SEM images revealed distinct morphological changes in the polymeric micelles containing the 2NB group before and after NIR‐laser irradiation. Before irradiation, the micelles exhibited a uniform, spherical structure with a smooth surface, indicating well‐defined self‐assembly. However, following NIR exposure, significant structural disruption was observed, characterized by deformation, increased porosity, and fragmentation of micelles.

The quantification of Ce6 release was conducted using UV spectroscopic analysis, revealing a pH‐dependent and laser‐enhanced drug release profile (Figure 1f,g). Over 48 h, less than 40% of Ce6 was released under physiological conditions (pH 7.4), ensuring minimal premature drug leakage in the systemic circulation. However, at tumor microenvironment pH (6.5), the release significantly increased to 60% ± 2.3%, while at lysosomal pH (5.5), it exceeded 72% ± 3.5% for mPCe6 micelles with laser irradiation. Similarly, mPNCe6 micelles exhibited a pronounced response to acidic conditions, with Ce6 release reaching 84% ± 3.2% at pH 5.5 upon NIR‐laser exposure. This trend suggests that the ester bond between Ce6 and mPEG‐2NB undergoes hydrolysis in the acidic lysosomal environment, leading to an accelerated drug release. Moreover, NIR‐induced photolysis of the 2NB group facilitated micelle disintegration, further enhancing Ce6 availability at the target site. These findings underscore the superior tumor‐specific activation and reduced systemic toxicity of the designed micellar system compared to conventional Ce6 formulations, making it a promising approach for controlled and efficient PDT. The hemolysis assay, a critical parameter for evaluating the blood compatibility of nanocarriers, demonstrated excellent biocompatibility of the developed micelles. As depicted in Figure 1h,i, the hemolysis percentage remained significantly low even at the highest tested concentration (500 μg/mL), with mPCe6 micelles inducing only 4.21% ± 0.32% hemolysis. In contrast, mPNCe6 micelles exhibited a slightly higher but minimal 4.54% ± 0.15% hemolysis. These values are well within the acceptable safety threshold of 5% per international hemocompatibility standards.

The results confirm that both micellar formulations pose minimal risk of erythrocyte membrane disruption, ensuring their suitability for IV administration. The low hemolysis percentage further reinforces the clinical translation potential of these formulations for safe and effective systemic delivery in photodynamic cancer therapy. Upon exposure to a NIR laser, mPCe6 Ms, mPNCe6 Ms, and a ROS probe, they underwent irradiation, as depicted in Figure S4. The increase in 7′‐dichlorodihydrofluorescein (DCF) absorption at 500 nm with extended irradiation time suggests a light dose‐dependent generation of ROS, even when these species are loaded onto mPCe6 Ms and mPNCe6 Ms. To assess the production of ^1^O_2_, crucial in photo‐oxidative damage induced by PDT, Ce6 in mPCe6 Ms and mPNCe6 Ms was evaluated by monitoring the reduction in DMA absorption at 378 nm. In the presence of ^1^O_2_, DMA transforms into a non‐fluorescent endoperoxide derivative. As anticipated, the solution containing mPCe6Ms, mPNCe6 Ms, and DMA, irradiated with a tungsten lamp at 660 nm (0.5 W) for up to 10 min, displayed a progressive decrease in DMA absorbance. This reduction is solely attributed to the ^1^O_2_ action generated upon Ce6 irradiation Figure S4A. mPNCe6 micelles generate more ^1^O_2_ by using the RNO and SOSG probe (Figure S4B,C).

### 4.3 Cellular internalization findings

**FIGURE 2:**
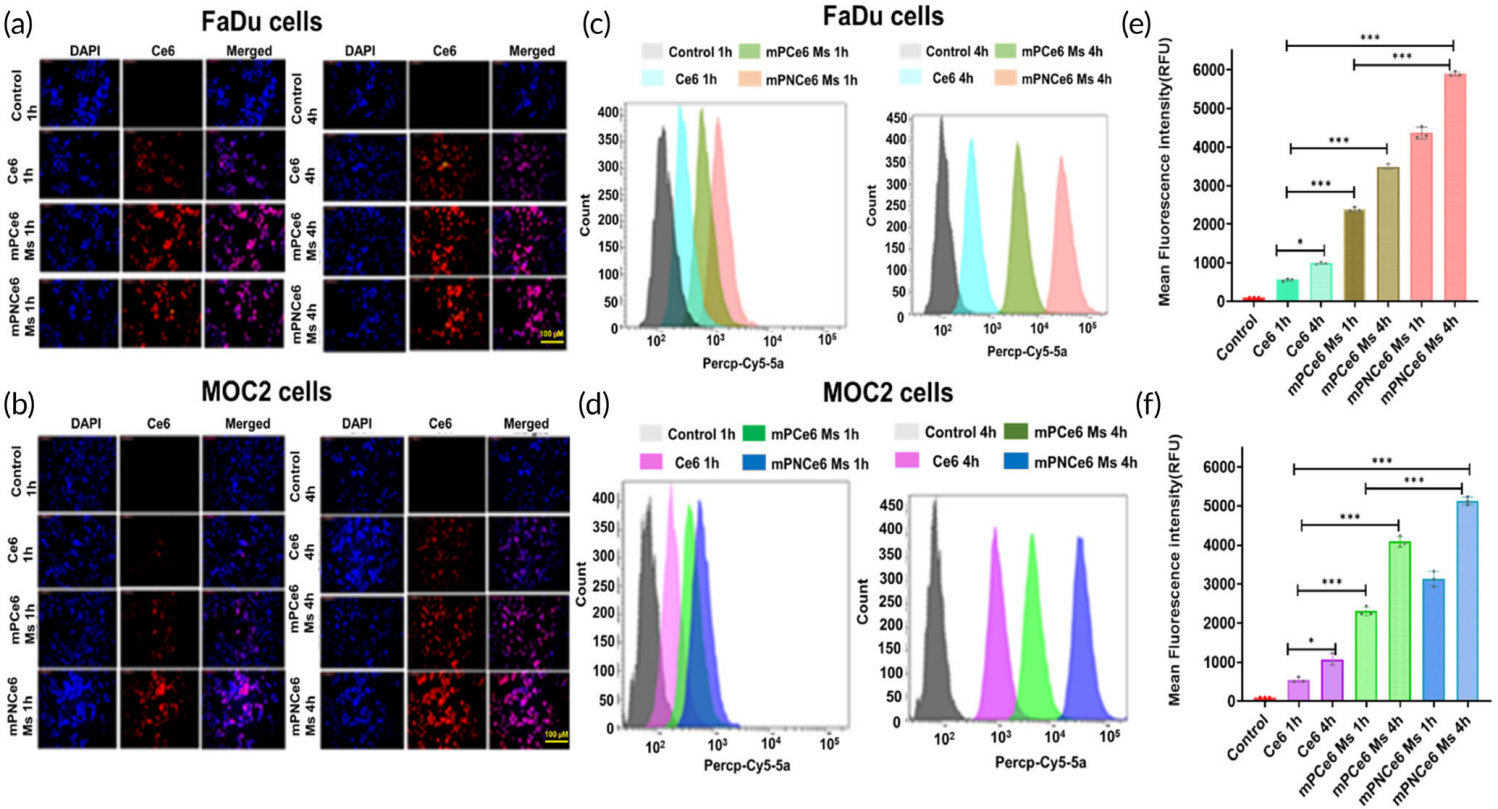
Cellular uptake and flow cytometry analysis of free chlorin e6 (Ce6), mPCe6, and PEGylated 2‐nitrobenzyl chlorin e6 (mPNCe6) micelles in FaDu and mouse oral squamous cell carcinoma cell lines (MOC2). (a and b) Fluorescence microscopy images showing the uptake of free Ce6, mPCe6, and mPNCe6 micelles by FaDu and MOC2 cell lines after 1‐ and 4‐h incubations at 37°C. Scale bar: 100 μm. (c and d) Flow cytometry analysis of free Ce6, mPCe6, and mPNCe6 micelles in FaDu (C) and MOC2 (D) cell lines, quantifying the relative cellular uptake of each formulation based on fluorescence intensity. (e and f) Graphical representation of flow cytometry data, highlighting the differences in uptake levels of free Ce6, mPCe6, and mPNCe6 micelles in both FaDu (e) and MOC2 (f) cell lines at different time points. *p < 0.05, ***p < 0.001.

According to the flow cytometry histogram, the fluorescence peak of mPNCe6 shifted to the right as the incubation time increased, indicating that mPNCe6 micelles internalization was more readily than free Ce6 (Figure 2). Fluorescence micrographs of cells treated with the formulations confirmed the findings. Free Ce6 showed a weak red signal intensity after 1 h of incubation, indicating low internalization. After 4 h of treatment, mPNCe6 micelle treated cells showed a much higher power of the red signal, followed by mPCe6 and finally free Ce6 in both cell lines (Figure 2a,b). At 4 h, the geo mean fluorescence of FaDu cells treated with Ce6, mPCe6, and mPNCe6 Ms was 1465 ± 63.25, 4236 ± 49.52, 5968 ± 80.65 (Figure 2c,e) and after 4 h incubation MOC2 cell treated with Ce6, mPCe6, and mPNCe6 Ms showed geo mean fluorescence 1142 ± 43.25, 3975 ± 60.32, and 4975 ± 34.21 (Figure 2d,f). The micelles' 2NB moiety undergoes photolysis upon NIR (660 nm) laser exposure, leading to structural destabilization and enhanced drug release at the tumor site. This phototriggered breakdown results in higher availability of Ce6 for uptake by both the cell lines compared to the mPCe6 and free Ce6.

### 4.4 In vitro cytotoxicity assessment

**FIGURE 3:**
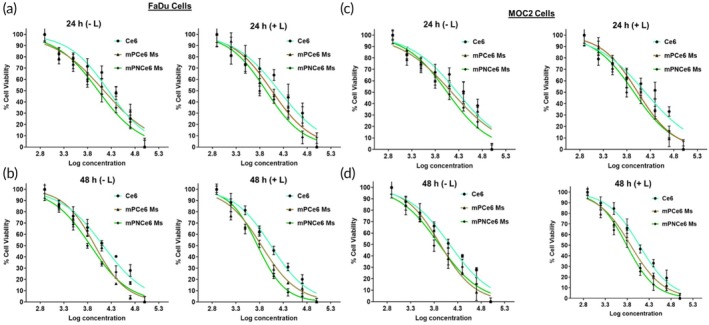
In vitro *cytotoxicity* evaluation of free chlorin e6 (Ce6), mPCe6 Ms, and PEGylated 2‐nitrobenzyl chlorin e6 (mPNCe6) Ms against FaDu and mouse oral squamous cell carcinoma cell lines (MOC2) under varying conditions. (a and b) Cytotoxicity of free Ce6, mPCe6 Ms, and mPNCe6 Ms against FaDu cells after 24‐ and 48‐h incubation, both with and without light irradiation (660 nm, 0.5 W/cm^2^ for 5 min). (c and d) Cytotoxicity analysis after 24‐ and 48‐h incubation of free Ce6, mPCe6 Ms, and mPNCe6 Ms in MOC2 cells, under similar conditions, comparing effects with and without irradiation.

In the MTT experiment conducted on the FaDu cell line, treating with free Ce6, mPCe6, and mPNCe6 Ms led to a concentration and time‐related decline in cell viability. Following irradiation, the IC50 values for Ce6, mPCe6, and mPNCe6 Ms against the FaDu cell line were 15.22, 11.25, and 8.52 μg/mL, respectively, after 24 h, while for the MOC2 cell line, the IC50 values were 15.41, 10.73, and 8.89 μg/mL, respectively (Table S1, Figure 3a,b). Similarly, after 48 h of irradiation, the IC50 values for Ce6, mPCe6, and mPNCe6 Ms were 10.91, 6.88, and 5.71 μg/mL, respectively, in the FaDu cell line, and 11.89, 7.52, and 6.12 μg/mL, respectively, in the MOC2 cell line (Figure 3c,d). Notably, the mPNCe6 Ms exhibited superior cell penetration, accumulating at higher Ce6 concentrations within the tumor cells, demonstrating enhanced cytotoxicity compared to free Ce6 across all concentrations in the cytotoxicity assay after laser irradiation. These formulations induced a more pronounced level of apoptosis relative to free Ce6, likely owing to the increased cellular uptake of Ce6 facilitated by the nanocarrier systems, resulting in a higher degree of apoptosis induction.

### 4.5 mPCe6 and mPNCe6 delivery improves in vitro efficacy

**FIGURE 4:**
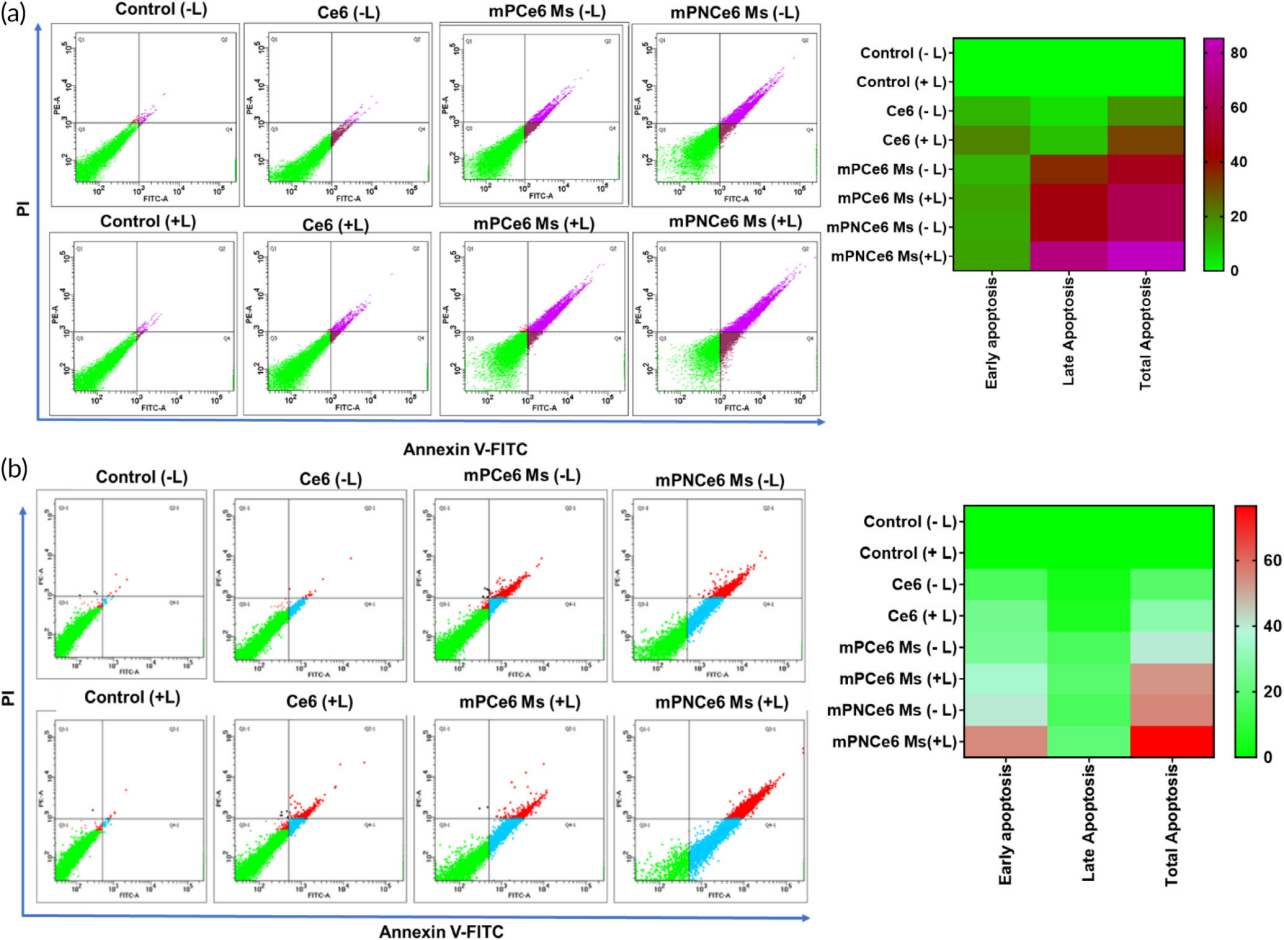
Apoptosis assay of cells using Annexin V/PI double staining method. (a and b) Apoptotic populations of FaDu and mouse oral squamous cell carcinoma cell line after exposure to different formulations: Culture medium, culture medium plus near‐infrared (NIR) irradiation, chlorin e6 (Ce6) solution, Ce6 solution plus NIR irradiation, mPCe6 Ms, mPCe6 Ms plus NIR irradiation, PEGylated 2‐nitrobenzyl chlorin e6 (mPNCe6) Ms, mPNCe6 Ms plus NIR irradiation. Cell counts (%) of necrotic cells (Q1), late apoptotic cells (Q2), early apoptotic cells (Q3), normal cells (Q4), and total apoptotic cells (Q2 and Q3) after different treatments. PI, propidium iodide.

The Annexin V assay evaluated the extent of apoptosis (programmed cell death) in response to treatment with free Ce6, mPCe6, and mPNCe6 micelles at a fixed concentration of 6 μg/mL of Ce6. As indicated in (Figure 4a,b) both cells treated with free Ce6 exhibited total apoptosis of 12.9% ± 3.5% in FaDu cells and 54.3% ± 4.5% for mPCe6 Ms, 82% ± 3.5% for mPNCe6 Ms, respectively, with laser irradiation. mPNCe6 Ms without laser exhibited total apoptosis of 58.8% ± 1.8%, whereas mPNCe6 Ms with laser produced total apoptosis of 75.3% ± 1.82% in MOC2 cell lines. When the cells undergo treatment, triggering the apoptotic pathway, phosphatidylserine (PS) relocates from the inner to the outer side of the plasma membrane, drawing in macrophages and instigating the creation of apoptotic bodies. Annexin V, acting as a distinct ligand for PS, selectively attaches to PS, a binding event detectable through its tagged fluorescence. Additionally, the assay employs PI, which binds specifically to necrotic cells.

**FIGURE 5:**
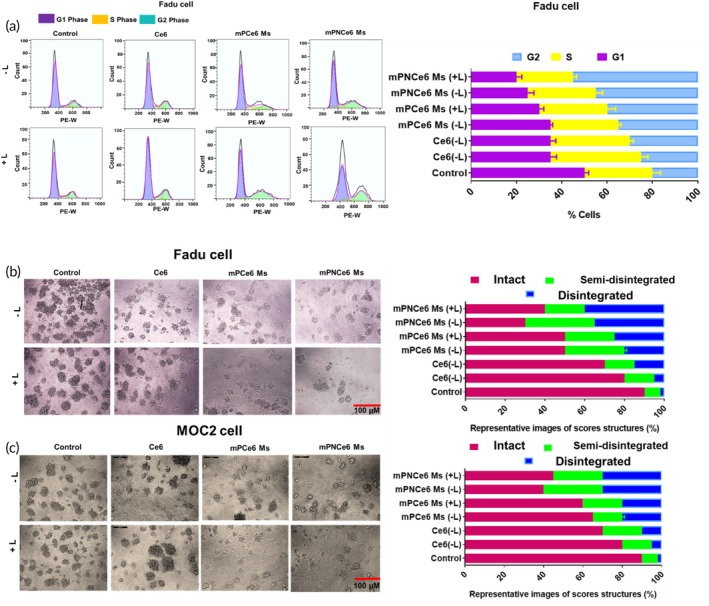
Cell cycle analysis and three‐dimensional (3D) tumor sphere assay to evaluate the effects of free chlorin e6 (Ce6), mPCe6 Ms, and PEGylated 2‐nitrobenzyl chlorin e6 (mPNCe6 Ms) on FaDu and mouse oral squamous cell carcinoma cell line (MOC2). (a) Cell cycle analysis of FaDu cells treated with free Ce6, mPCe6 Ms, and mPNCe6 Ms. The analysis demonstrates the distribution of FaDu cells in different phases of the cell cycle (G0/G1, S, and G2/M) after treatment. (b and c) 3D tumor sphere (memosphere) assay of free Ce6, mPCe6 Ms, and mPNCe6 Ms on FaDu and MOC2 cell lines respectively, with and without laser irradiation. The assay evaluates the effects of these formulations on the growth and viability of 3D tumor spheroids, providing insights into the efficacy of photodynamic therapy under laser irradiation (660 nm).

The cell cycle analysis data revealed that mPNCe6 NPs arrest the G2/M phase compared to the other formulation (Figure 5a). To assess the impact of micelles on tumor development and advancement in a setting that mirrors the tumor microenvironment more closely, we grew two oral cancer cell lines as 3D spheres in Matrigel. Both levels of micelles prompted varying degrees of disruption in the cell spheres, with higher drug concentrations leading to more pronounced structural breakdown in the two oral cancer cell lines (Figure 5b,c). These findings indicate that micelles hold considerable promise for substantially restraining oral cancer cell lines.

**FIGURE 6:**
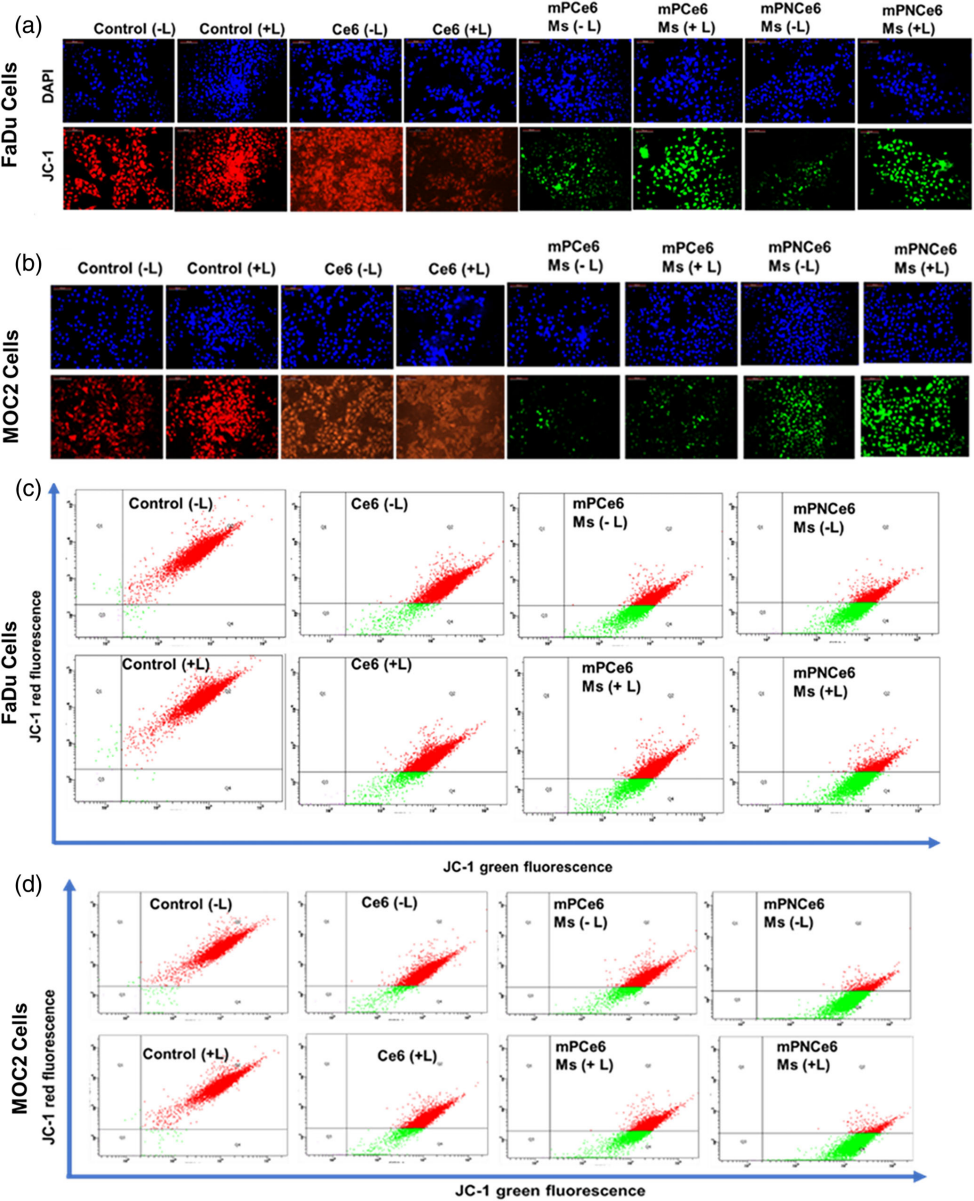
Evaluation of mitochondrial membrane potential in FaDu and mouse oral squamous cell carcinoma cell line (MOC2) cells across different treatment groups. (a and b) Fluorescence microscopy analysis of mitochondrial membrane potential in FaDu (a) and MOC2 (b) cells treated with different formulations, including free chlorin e6 (Ce6), mPCe6 Ms, and PEGylated 2‐nitrobenzyl chlorin e6 (mPNCe6) Ms. The changes in fluorescence intensity indicate the disruption or preservation of mitochondrial membrane potential, serving as an indicator of mitochondrial health and apoptosis induction. (c and d) Flow cytometry analysis of mitochondrial membrane potential in FaDu (c) and MOC2 (d) cells for the same treatment groups.

Intracellular cytotoxic ROS can damage cells' mitochondria, resulting in cell death. Our findings showed that mPNCe6 Ms is an effective drug delivery mechanism for Ce6. In our investigation, the mitochondrial membrane potential of our mPNCe6 Ms‐treated formulation group showed more severe collapse than mPCe6 and free Ce6. High levels of ROS can osmotically inflate the mitochondrial matrix and disrupt the mitochondrial permeability transition pore, compromising the integrity of the mitochondrial membrane and causing the mitochondrial transmembrane potential to dissipate fast. The fluorescence images of both cells treated with free Ce6, mPCe6, and mPNCe6 micelles and tagged with JC‐1 dye are shown in (Figure 6a,b). The dye exhibits red fluorescence for control cells, an intermediate between green and red fluorescence for free Ce6, and a bright green fluorescence for mPNCe6 Ms. According to the findings, treating FaDu and MOC2 cells with mPNCe6 Ms disrupted mitochondrial membrane potential. Furthermore, mitochondrial dysfunction could be implicated in the mPNCe6 micelles‐induced apoptosis process.

**FIGURE 7:**
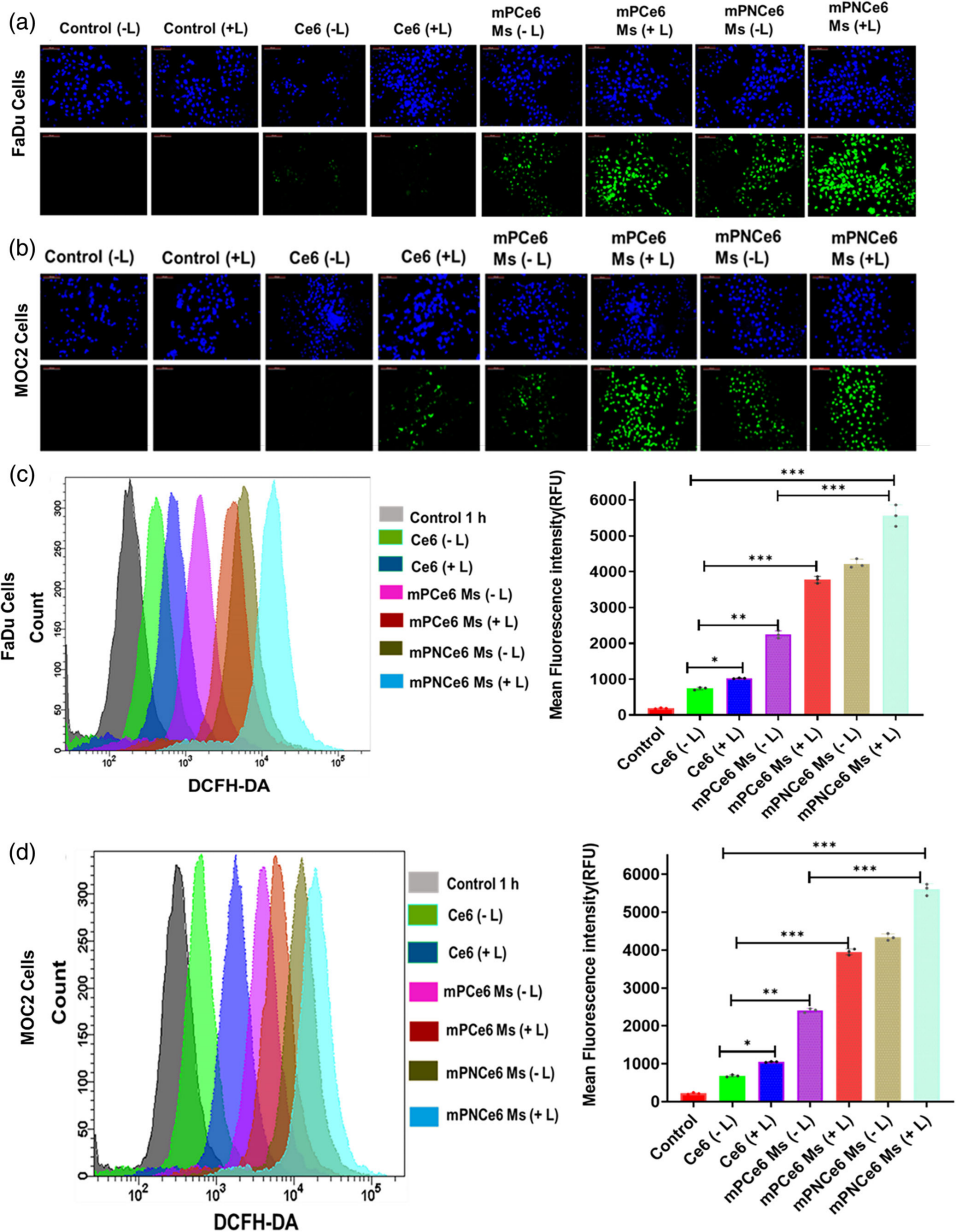
Determination of intracellular reactive oxygen species (ROS) generation in FaDu and mouse oral squamous cell carcinoma cell line (MOC2) cells using the DCFH‐DA assay. (a and b) Fluorescence microscopy images showing ROS generation in FaDu (a) and MOC2 (b) cells treated with free chlorin e6 (Ce6), mPCe6 Ms, and PEGylated 2‐nitrobenzyl chlorin e6 (mPNCe6) Ms, both with and without laser irradiation. The increased fluorescence intensity in treated cells under laser irradiation (660 nm) indicates enhanced ROS production, highlighting the photodynamic activity of the formulations. (c and d) Quantitative analysis of fluorescence intensity using flow cytometry in FaDu (c) and MOC2 (d) cells, providing a comparative assessment of ROS levels across different treatment groups. The probability value (*p*‐value) of  < 0.05,  < 0.01, and  < 0.001 represents statistically significant data as *, **, and ***.

Afterward, the ability of ROS generation of mPCe6 and mPNCe6 Ms in FaDu cells was measured using the cell‐permeable fluorescent probe DCFH‐DA. As per Figure 7a,b, mPCe6 and mPNCe6 Ms, the group with laser irradiation resulted in abundant ROS generation inside both cells. Compared to other control groups except Ce6, they showed solid green fluorescence, specifying the efficient Ce6 by mPNCe6 Ms and the excellent ability to generate the ROS inside the cells with the assistance of a laser. Based on the above findings, the mPNCe6 Ms could enter into cells, resulting in cancer cell apoptosis by ROS generation with laser irradiation.

### 4.6 mPCe6 and mPNCe6 micelles induce solid tumor regression

**FIGURE 8:**
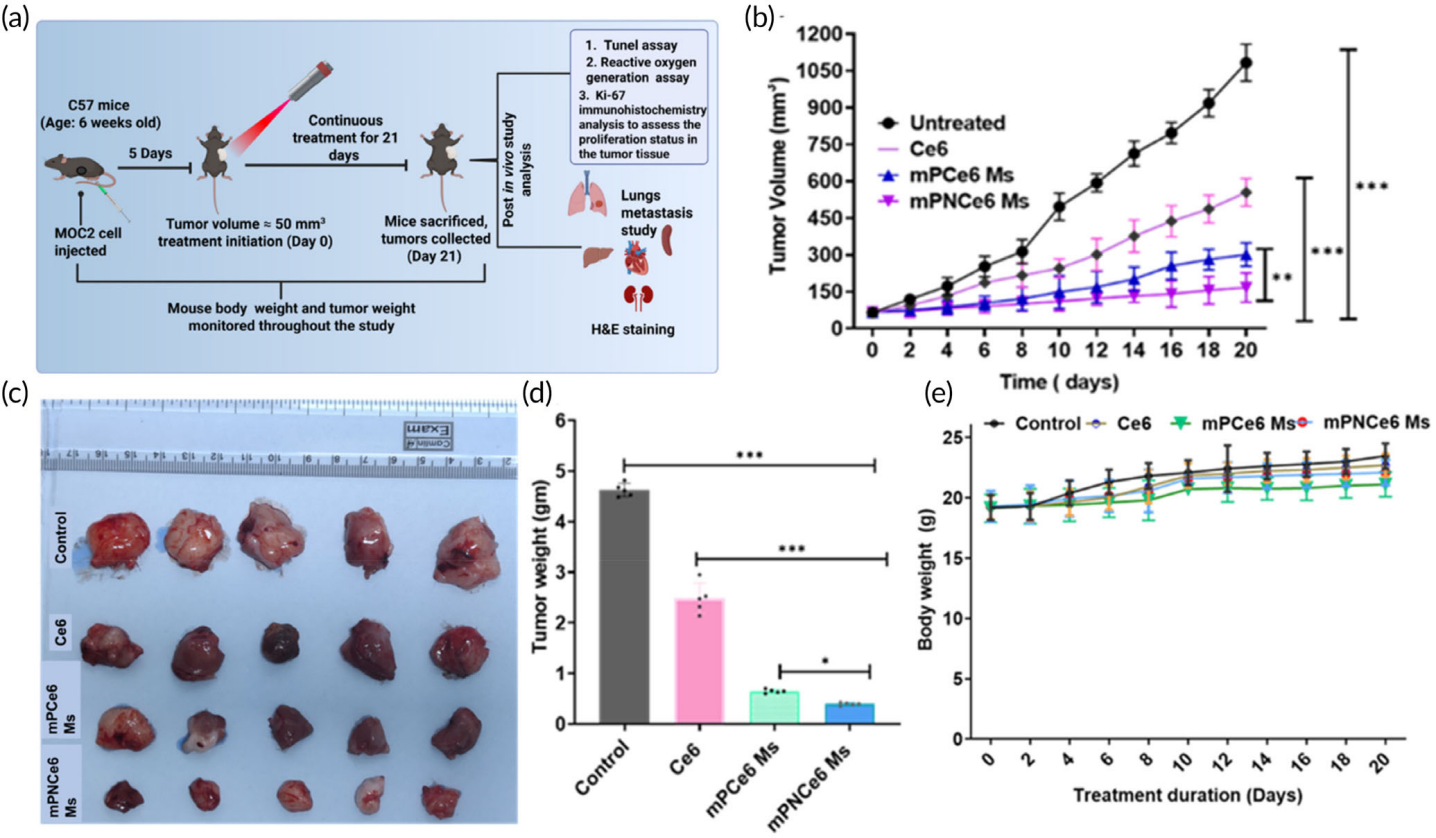
Evaluation of in vivo antitumor therapeutic efficacy of free chlorin e6 (Ce6), mPCe6 Ms, and PEGylated 2‐nitrobenzyl chlorin e6 (mPNCe6) Ms in the mouse oral squamous cell carcinoma cell line (MOC2) tumor‐bearing mouse model. (a) Schematic representation of the experimental protocol outlining the treatment regimen for evaluating the in vivo antitumor efficacy. MOC2 tumor‐bearing mice were treated with free Ce6, mPCe6 Ms, and mPNCe6 Ms with laser irradiation (660 nm) over a specified period. The timeline includes tumor implantation, treatment schedule, and laser irradiation details. (b) Tumor volume inhibition assay compares tumor growth rates in different treatment groups over time. (c) Graphical representation of MOC2 tumor‐bearing mice in various treatment groups, providing a visual overview of tumor size and progression during the experiment. (d) The measurement of tumor weight in MOC2 tumor‐bearing mice after treatment demonstrates the reduction in tumor mass following different treatments. (e) Body weight analysis of MOC2 tumor‐bearing mice throughout the study, assessing the overall health and potential toxicity of the treatments. The probability value (*p*‐value) of  < 0.05,  < 0.01, and  < 0.001 represents statistically significant data as *, **, and ***.

Following the in vitro therapeutic efficacy, we investigated the in vivo anticancer efficiency of Ce6, mPCe6 and mPNCe6 Ms (Figure 8a). During the experiment, the PBS, Ce6, mPCe6, and mPNCe6 Ms were intravenously administered to the mice with MOC2 tumors (initial tumor volume of 70 mm^3^). The group treated with PBS showed a tumor volume of 1045.6 ± 42.1 mm^3^ after 21 days, indicating the well‐established rapid growth of tumors in mice (Figure 8b). Even though the MOC2 tumors showed a slight reduction in tumor growth in the first 10 days for all the samples, the tumor volume increased from the point of cessation in the case of Ce6 treatment (451.21 ± 11.4), mPCe6 (285.3 ± 11.32 mm^3^), and mPNCe6 Ms (135.3 ± 7.02 mm^3^) within 21 days (Figure 8c). The average tumor weight in mice administered control, free Ce6, mPCe6, and mPNCe6 Ms groups was found to be 3.87 ± 0.11, 2.28 ± 0.14, 0.30 ± 0.08, and 0.20 ± 0.04 g, respectively, as shown in Figure 8d, and a modest increase was found in the body weight of mice throughout the experiment period (Figure 8e).

#### 4.6.1 In vivo biodistribution study of mPCe6 mPNCe6 micelles

**FIGURE 9:**
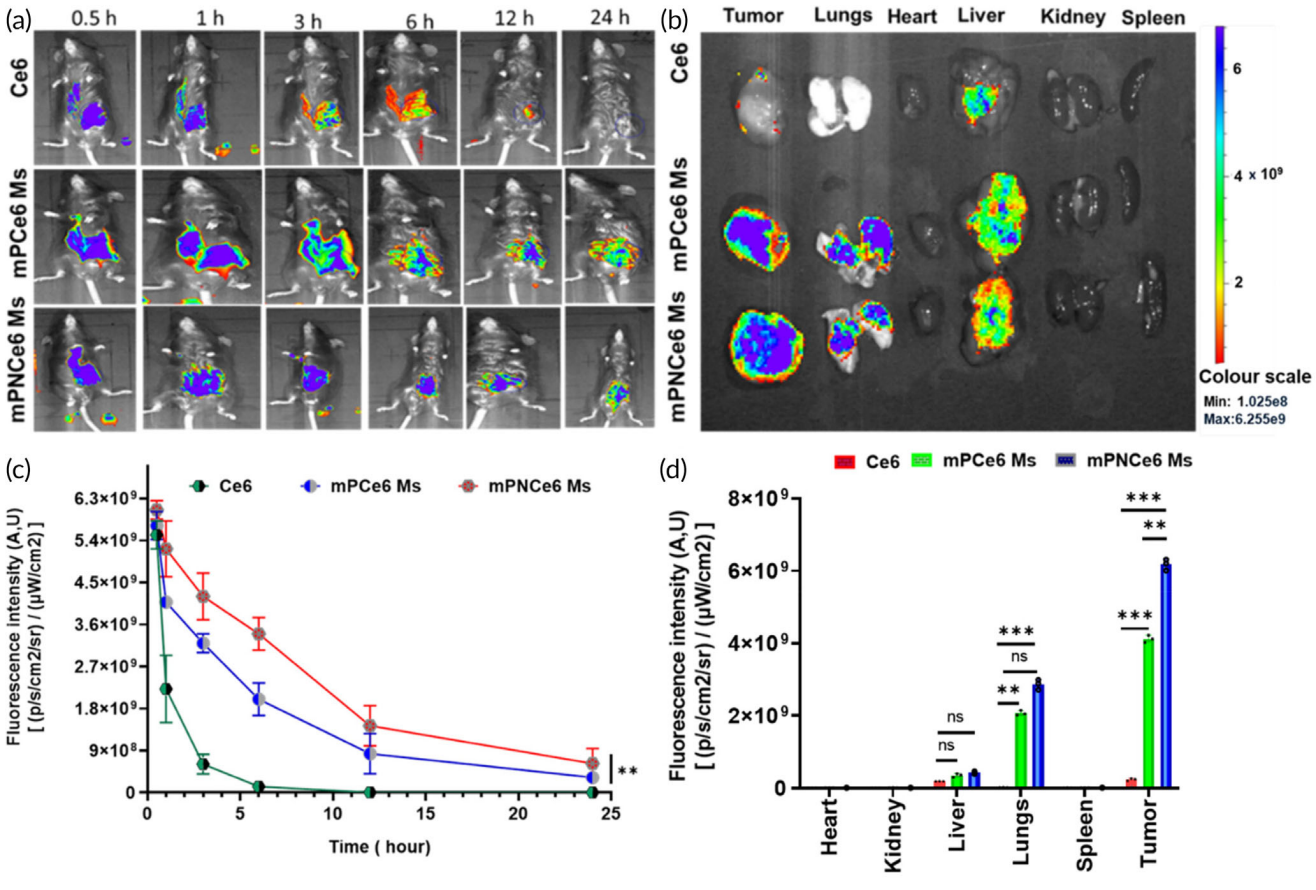
In vivo biodistribution study of chlorin e6 (Ce6), mPCe6 Ms, and PEGylated 2‐nitrobenzyl chlorin e6 (mPNCe6) Ms in mouse oral squamous cell carcinoma cell line (MOC2) tumor‐bearing mice. (a) Biodistribution analysis of free Ce6, mPCe6 Ms, and mPNCe6 Ms in MOC2 tumor‐bearing mice, tracked at different time points post‐administration. Fluorescence imaging highlights the accumulation of each formulation in various organs, including the tumor, liver, spleen, kidneys, and lungs. (b) Comparative analysis of fluorescence intensity in the tumor tissue versus other organs, demonstrating the enhanced tumor‐targeting efficiency of mPNCe6 Ms compared to free Ce6 and mPCe6 Ms. (c) Quantitative fluorescence data of the whole body biodistribution. (d) Quantitative fluorescence data of organs (heart, kidney, liver, lungs spleen, and tumor). The probability value (*p*‐value) of  < 0.05,  < 0.01, and  < 0.001 represents statistically significant data as *, **, and ***.

Live imaging biodistribution studies of excretory organs of animals revealed a non‐specific distribution of mPCe6, mPNCe6 Ms, and it cleared off more quickly than Ce6 deposited in the tumor (Figure 9a,c). Fluorescence analysis by ex vivo for the tumor tissue and dissected organs such as kidney, lung, spleen, liver, and heart after 36 h of injection demonstrated a significant increase in the accumulation of mPCe6, and mPNCe6 Ms significantly increased tumor accumulation compared to free Ce6 (Figure 9c,d). The increased accumulation of mPCe6 and mPNCe6 micelles in the tumor tissue compared to free Ce6 can be attributed to their EPR effect. Due to their nanoparticulate size and formulation, the micelles can more efficiently penetrate tumor tissues and accumulate within the tumor microenvironment, which has leaky vasculature.

### 4.7 Histological findings

**FIGURE 10:**
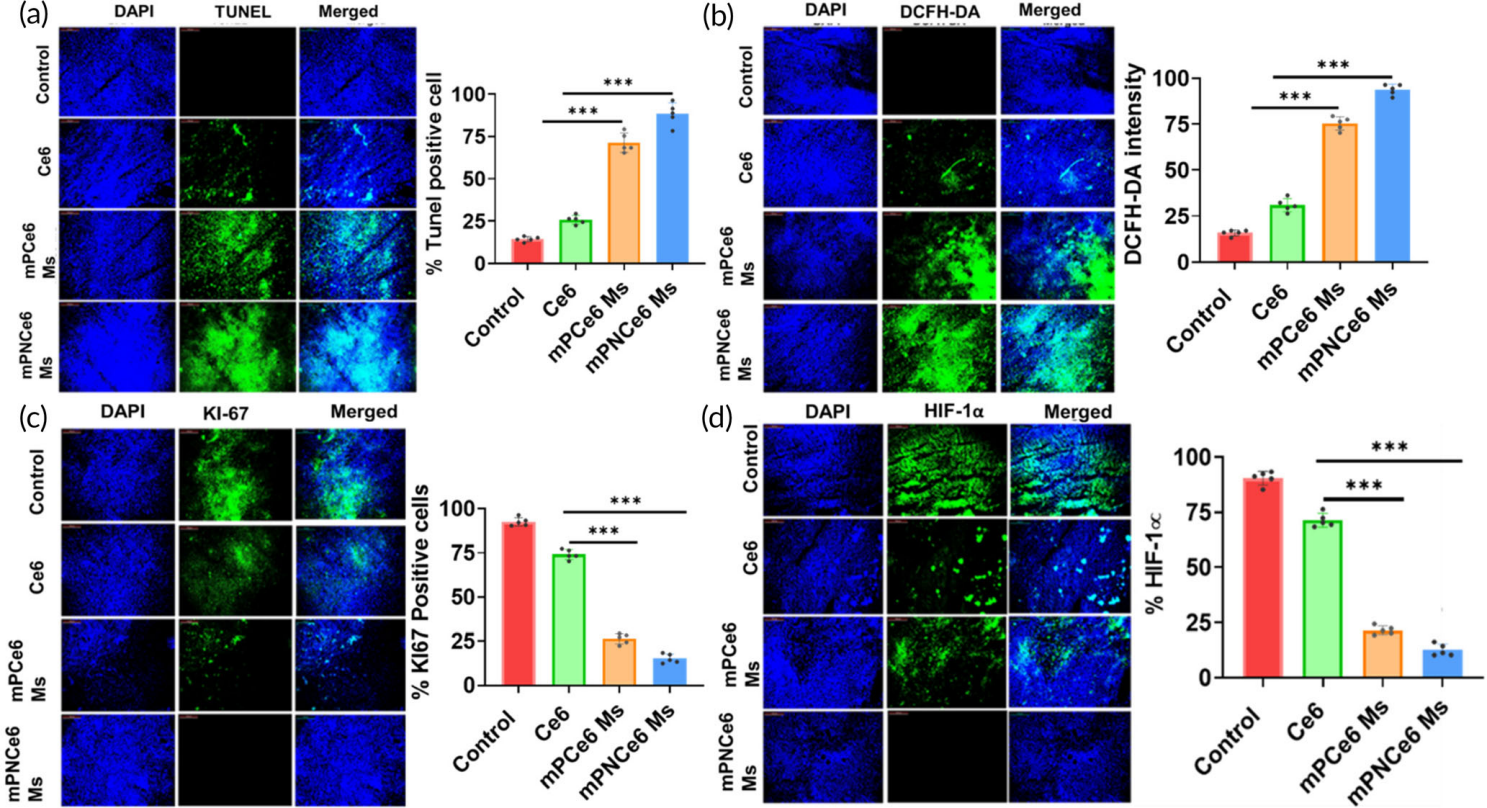
*Immunohistochemistry* analysis of tumor sections from MOC2 tumor‐bearing mice treated with free chlorin e6 (Ce6), mPCe6 Ms, and PEGylated 2‐nitrobenzyl chlorin e6 (mPNCe6) Ms. (a) Representative images of the TUNEL assay in tumor sections, indicating apoptotic cell death. Positive TUNEL staining highlights DNA fragmentation in apoptotic cells, demonstrating the effectiveness of each treatment in inducing apoptosis within the tumor tissue. (b) ROS generation assay showing the presence of reactive oxygen species (ROS) in tumor sections, detected by fluorescence staining. Increased ROS levels in the mPNCe6 Ms‐treated group, particularly after laser irradiation, indicate enhanced oxidative stress and potential tumor cell damage from photodynamic therapy. (c) Ki67 staining to assess cellular proliferation in the tumor sections. The Ki67‐positive cells represent actively dividing tumor cells, with reduced Ki67 expression in treated groups suggesting inhibited tumor growth and proliferation, especially in the mPNCe6 Ms + laser group. (d) Hypoxia‐inducible factor 1‐alpha (HIF‐1α) staining in tumor sections to evaluate the hypoxic status of the tumor microenvironment. Decreased HIF‐1α expression in treated tumors suggests a reduction in tumor hypoxia, which may contribute to the improved therapeutic outcomes observed with mPNCe6 Ms. DAPI, 4,6‐diamidino‐2‐phenylindole. The probability value (*p*‐value) of  < 0.05,  < 0.01, and  < 0.001 represents statistically significant data as *, **, and ***.

The mice treated with mPNCe6 displayed the most intense green fluorescence when assessed by the DCFH‐DA probe, a tool used to gauge oxidative stress levels (Figure 10b). Once internalized into cells, the dye undergoes oxidation via intracellular ROS, creating DCF and subsequent emission of green fluorescence. Chemotherapy and photodynamic therapy (PDT) induce the generation of various ROS, such as superoxide anion, hydrogen peroxide, and hydroxyl radicals, causing damage to cancer cells. The nanoformulations triggered a higher production of ROS within tumor tissues compared to administering the unbound Ce6 medication. PDT primarily eradicates cancer cells by significantly increasing ROS production. The study revealed that mPNCe6 demonstrated a four‐fold increase in ROS generation compared to all other examined formulations. This therapy heightened oxidative stress, leading to increased apoptosis in cancer cells, evidenced by TUNEL‐positive cells emitting green fluorescence (Figure 10a). Cells treated with the nanoformulation exhibited higher TUNEL‐positive cell expression than those treated with free medicines, with mPNCe6 showing the highest population of apoptotic cells among the nanoformulations. Furthermore, analysis of tumor sections aimed to identify Ki‐67 expression, a proliferation marker (Figure 10c). Untreated tumor tissue displayed notably elevated Ki‐67 expression, indicating rapid cell division and potential metastatic capacity. Conversely, the formulations exhibited reduced signals, consistent with ROS and TUNEL results, suggesting their ability to generate ROS, induce apoptosis, and suppress cell growth rates. The hypoxia‐inducible factor 1‐alpha (HIF‐1α) significantly decreased after the mPNCe6 treatment, indicating our formulation showed more therapeutic efficacy than the free drug (Figure 10d).

### 4.8 Lungs metastasis

**FIGURE 11:**
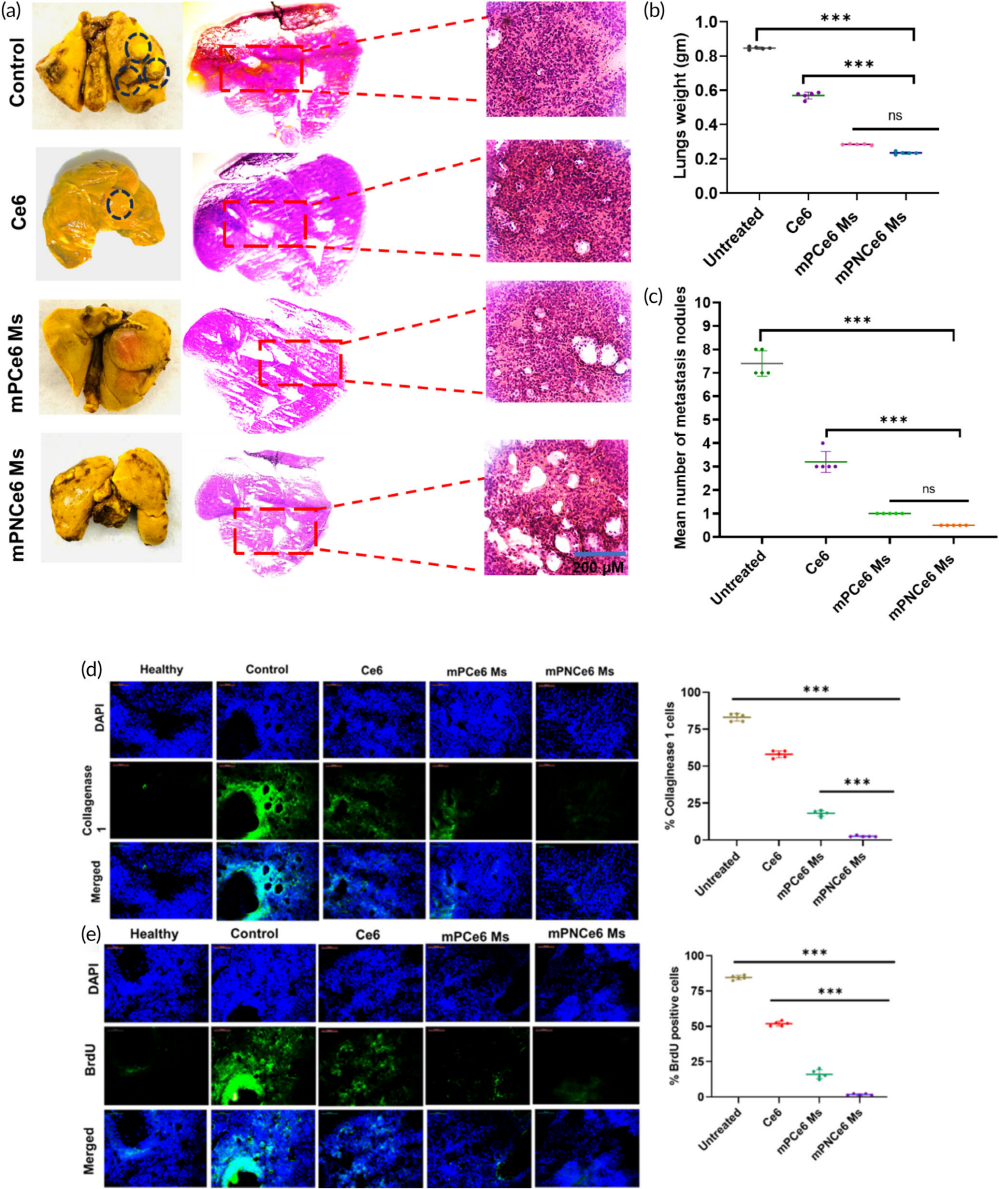
(a) Hematoxylin and eosin staining of lung sections demonstrating higher cellular proliferation in control groups compared to treatment groups. (b) Bar graph representing the lung weights across different groups, indicating reduced metastasis in treatment groups. (c) Quantification of lung nodules in each group, showing fewer metastatic nodules in treated animals. (d) Detection of proliferating metastatic cells in lung tissue through BrdU incorporation. BrdU was administered via tail vein injection, and 2 h post‐injection, the lungs were resected and stained with anti‐BrdU antibody to visualize proliferating cells. (e) Collagen I staining of lung sections to assess collagen levels and potential fibrosis, highlighting differences between control and treatment groups. Ce6, chlorin e6; DAPI, 4,6‐diamidino‐2‐phenylindole; mPNCe6, PEGylated 2‐nitrobenzyl chlorin e6. The probability value (*p*‐value) of <0.05,  < 0.01, and  < 0.001 represents statistically significant data as *, **, and ***.

This difference likely stems from cancer cells becoming trapped within blood vessels, facilitating the formation of metastatic lung regions. Another critical observation was the presence of lung metastasis upon the mice's death (Figure 11a). Throughout the experiment, lung weights were recorded, with saline‐treated, Ce6‐treated, mPCe6‐treated, and mPNCe6‐treated groups showing average lung weights of 0.85 ± 0.12, 0.62 ± 0.14, 0.24 ± 0.08, and 0.21 ± 0.07 g, respectively (Figure 11b). Furthermore, treatment with mPNCe6 significantly reduced the mean number of lung nodules (Figure 11c). BrdU (Bromodeoxyuridine), a thymidine analog, plays a crucial role in BrdU assays by marking proliferating cells. Microscopic examination of BrdU‐stained lung sections revealed a significant finding: the number of endothelial cells was considerably higher in metastasized lungs of both Ce6‐treated and untreated groups compared to those treated with mPCe6 and mPNCe6 micelles (Figure 11d). Additionally, collagen levels were notably lower in the mPCe6 and mPNCe6‐treated groups compared to the untreated group (Figure 11e).

### 4.9 Safe and well‐tolerated mPCe6 and mPNCe6 micelle therapy in mice

**FIGURE 12:**
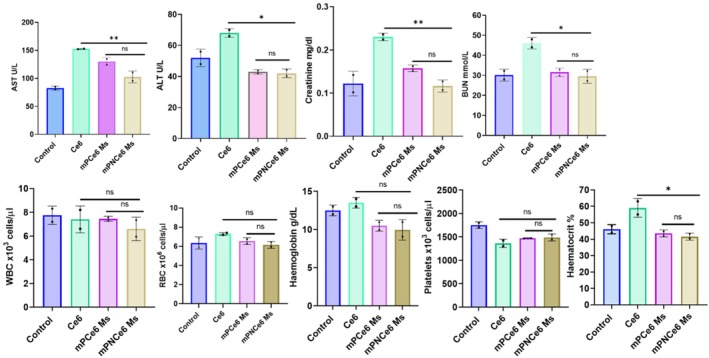
Blood biochemistry analysis of chlorin e6 (Ce6), mPCe6 Ms, and PEGylated 2‐nitrobenzyl chlorin e6 (mPNCe6) Ms. ALT, alanine aminotransferase. The probability value (*p*‐value) of  < 0.05,  < 0.01, and  < 0.001 represents statistically significant data as *, **, and ***.

While considering photodynamic therapy, it has the advantage of being minimally invasive and avoids several limitations associated with conventional treatments such as radiation, chemotherapy, and surgery. In our study, the safety of the developed photodynamic therapy was evaluated by analyzing key blood biochemical markers, including BUN (Blood Urea Nitrogen), creatinine, alanine aminotransferase (ALT), alkaline phosphatase (ALP), and total proteins (Figure 12). The assessment confirmed that all measured serum parameters remained within normal physiological ranges, suggesting that the IV formulations were non‐toxic to major organs such as the liver and kidneys. It is important to note that the blood biochemistry analysis was conducted using *n* = 2 mice per group, which limits the statistical power and precludes definitive conclusions regarding systemic toxicity. This small sample size was due to the limited availability of biological material and was intended solely as a preliminary safety assessment. We acknowledge this limitation and recommend that future studies include larger cohorts to confirm these findings. All other in vivo experiments reported in this study, including therapeutic efficacy and biodistribution studies, were conducted using *n* = 5 and *n* = 3 mice per group to ensure sufficient statistical reliability.

## 5 CONCLUSION

Here, a novel drug photo‐delivery system based on NIR light triggered photo‐cleavage of a 2‐NB linker, binding to the methoxy polyethylene glycol, was coupled with Ce6 to create nano micelles The prepared nanodevice released a conjugated (Ce6) light at low power irradiance upon irradiation with 660 nm light due to the multiphoton absorption and dissociation mediated by the 2NB. The conjugated micelles functioned as carriers for Ce6. The polymeric micelles exhibited stability at a temperature of 4°C, displayed ideal physicochemical properties regarding their spherical size, and had low CMC. The micelles entered the FaDu and MOC2 cells over time, resulting in a considerably greater cell death than free Ce6 or mPCe6 micelles. The findings revealed that the micelles exhibited a remarkable efficacy in triggering apoptosis, DNA damage, ROS formation, and mitochondrial membrane depolarization, ultimately leading to a greater extent of cellular demise than unbound Ce6. The in vivo therapeutic investigations conducted on mice bearing MOC2 tumors demonstrated that mPNCe6 micelles induced noteworthy tumor growth inhibition, reduced lung metastasis, and alleviated toxicity. Analysis of serum and blood components indicated minimal systemic toxicity associated with mPNCe6 micelles. Therefore, the developed photo‐activatable and degradable nano platform holds promise as a potential nanomedicine to treat oral cancer, mitigate conventional toxicity, and prevent metastasis.

## AUTHOR CONTRIBUTIONS

**Milan Paul**: Conceptualization, methodology, formal analysis and investigation, writing—original draft preparation, writing—review and editing. **Swati Biswas**: Conceptualization, methodology, formal analysis and investigation, writing—review and editing, and funding acquisition. All authors read and approved the final manuscript.

## CONFLICT OF INTEREST STATEMENT

The authors have no relevant financial or non‐financial interests to disclose.

## Supporting information


**Data S1.** Supporting Information.

## Data Availability

The data supporting the findings of this study are available from the corresponding author upon reasonable request.
