# Supplementary material for: Near Infrared‐light responsive chlorin e6 pro‐drug micellar photodynamic therapy for oral cancer
Source: Bioeng Transl Med. 2025 Sep 16;10(6):e70036. doi: 10.1002/btm2.70036 (PMC12617552; doi:10.1002/btm2.70036)
Supplement: Supplementary file 1 — Data S1. Supporting Information. [file BTM2-10-e70036-s001.docx]

**Supplementary Information**

**NIR-Light Responsive Chlorin e6 Pro-Drug Micellar Photodynamic Therapy for Oral Cancer**

Milan Paul, Swati Biswas*

Nanomedicine Research Laboratory, Department of Pharmacy, Birla Institute of Technology & Science-Pilani, Hyderabad Campus, Jawahar Nagar, Medchal, Hyderabad-500078, Telangana, India

*Corresponding author

Swati Biswas, Ph.D

Nanomedicine Research Laboratory

Professor

Department of Pharmacy

BITS-Pilani, Hyderabad campus

Medchal, Hyderabad-500078

Telangana, India

Email. swati.biswas@hyderabad.bits-pilani.ac.in

Phone. (+91)-40-6630-3630

Fax. (+91)-40-6630-3998


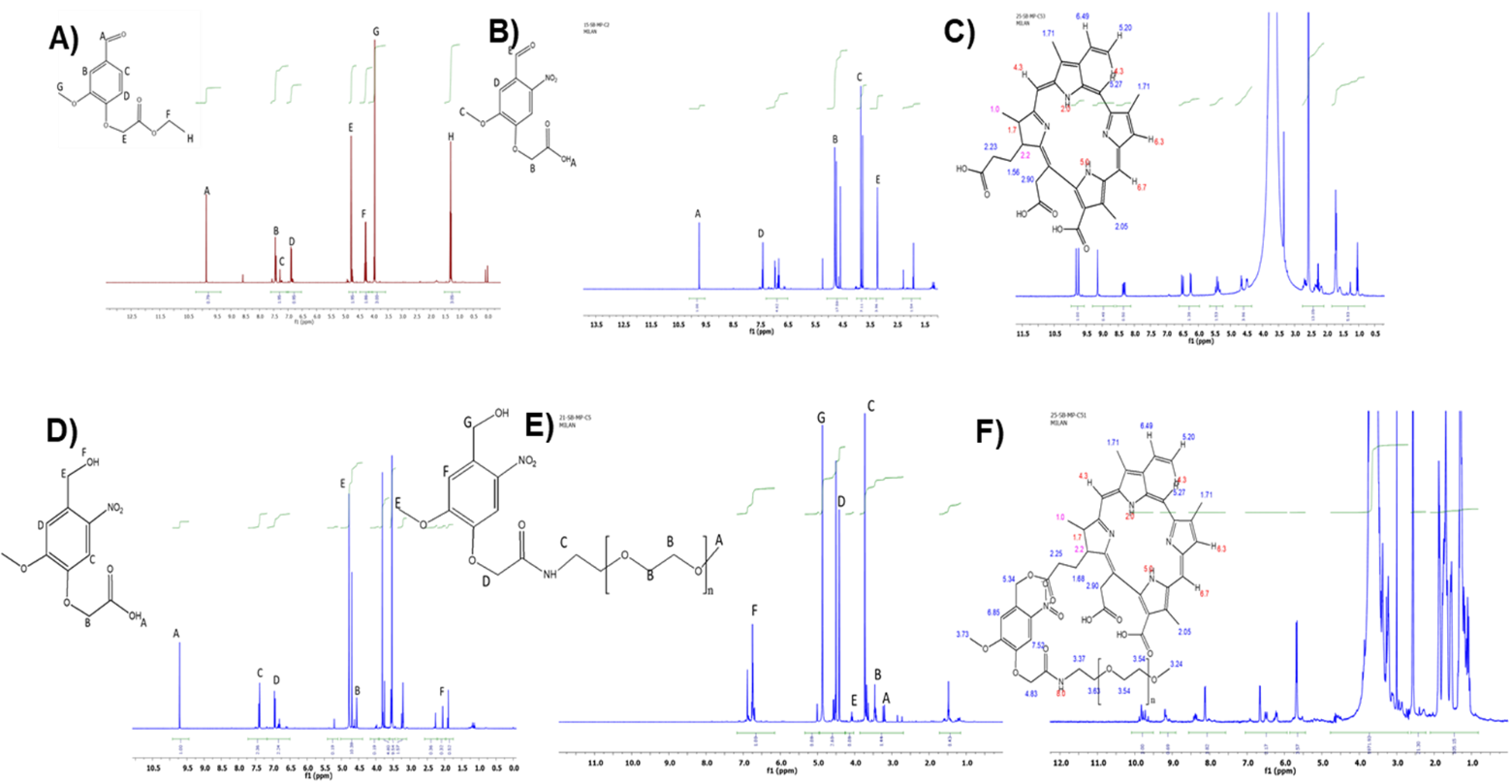


**Figure S1.** ^1^H NMR spectra of Ethyl 2-(4-formyl-2-methoxyphenoxy) acetate (A); 2-(4-formyl- 2-methoxy-5-nitrophenoxy) acetic acid (B); Ce6 (C); 2-(4-hydroxymethyl)-2-methoxy-5-nitrophenoxy) acetic acid (D); PEG-NH 2-(4-hydroxymethyl)-2-methoxy-5-nitrophenoxy) (E); mPEG-2NB-Ce6 (F).

**
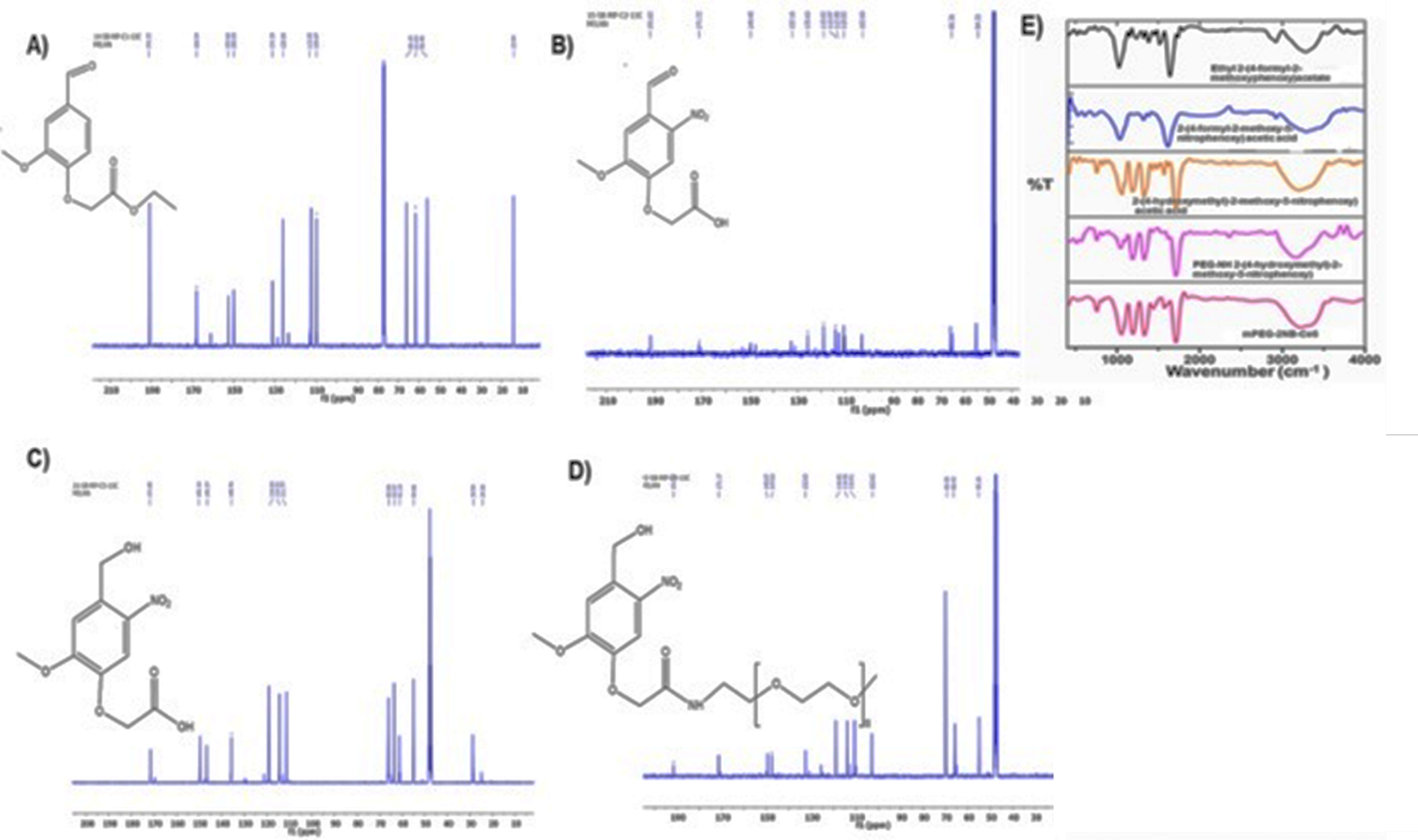
**

**Figure S2.** ^13^C NMR spectra of Ethyl 2-(4-formyl-2-methoxyphenoxy) acetate (A); 2-(4-formyl- 2-methoxy-5-nitrophenoxy) acetic acid (B); 2-(4-hydroxymethyl)-2-methoxy-5-nitrophenoxy) acetic acid (D); PEG-NH 2-(4-hydroxymethyl)-2-methoxy-5-nitrophenoxy) (D); FT-IR spectra of Ethyl 2-(4-formyl-2-methoxyphenoxy) acetate, 2-(4-formyl-2- methoxy-5-nitrophenoxy) acetic acid, 2-(4-hydroxymethyl)-2-methoxy-5-nitrophenoxy) acetic acid, PEG-NH 2-(4-hydroxymethyl)-2-methoxy-5-nitrophenoxy), mPEG-2NB-Ce6 (E);

**
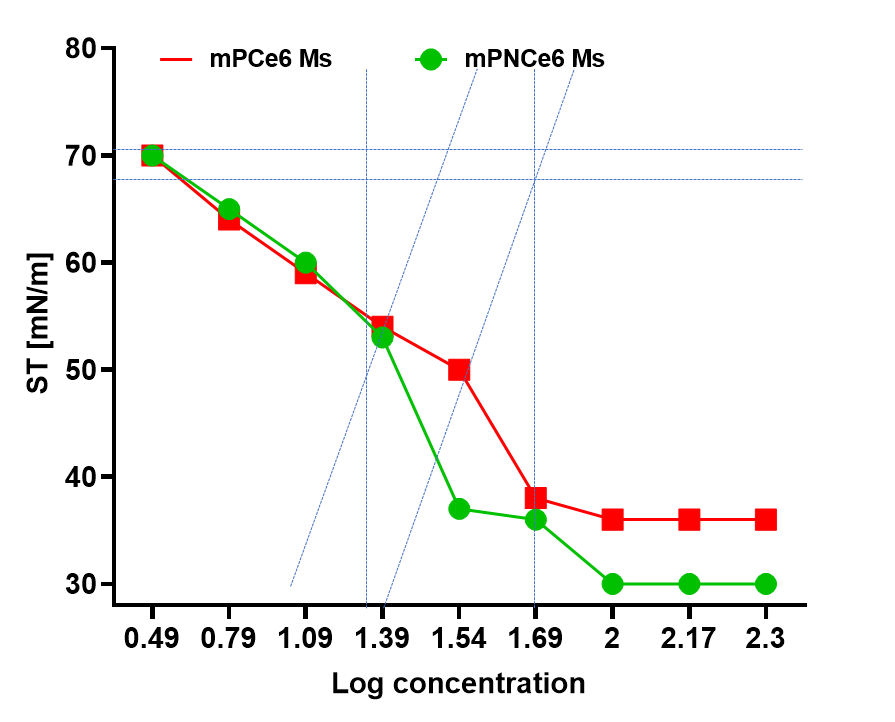
**

**Figure S3.** CMC analysis by surface tension method of mPCe6 and mPNCe6 MS


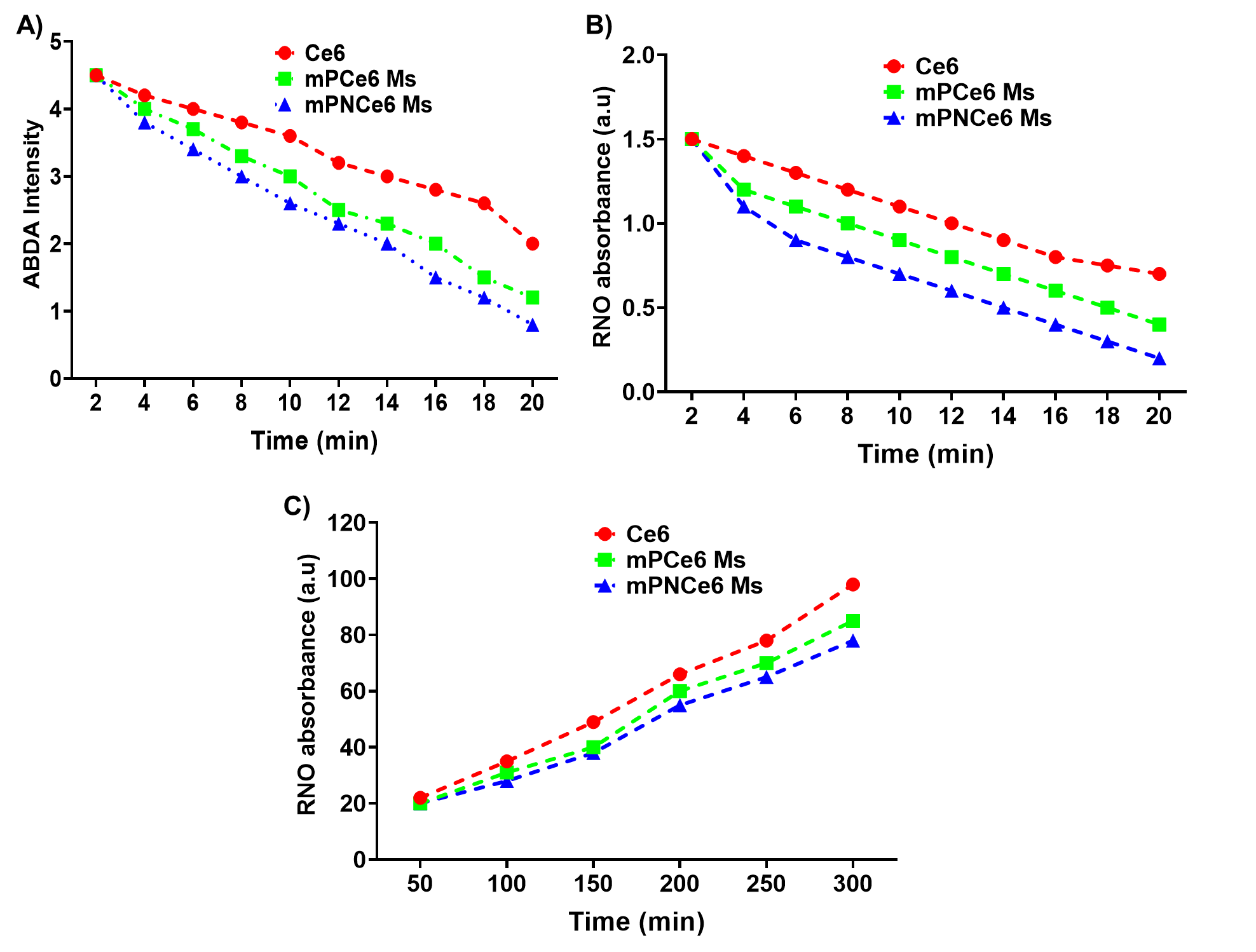


**Figure S4.** Change in the fluorescence intensity of DMA (ex 360 nm; em 436 nm) concerning time in the presence of SOG by mPCe6, and mPNCe6 Ms, and free Ce6 in DMSO, respectively (A); Time-dependent photobleaching of RNO by free Ce6 and mPCe6, and mPNCe6 Ms, (B); Changes in fluorescence intensity of SOSG in the presence of free Ce6 and mPCe6, and mPNCe6 Ms in distilled water(C).

**Table S1: IC50 value of free Ce6, mPCE6 Ms, mPNCe6 Ms in FaDu and MOC2 cell lines**

***FaDu***

| ***IC50 (µg/ml)*** | ***Free Ce6*** | | ***mPCe6 Ms*** | | ***mPNCe6 Ms*** | |
| --- | --- | --- | --- | --- | --- | --- |
|  | ***-L*** | ***+L*** | ***-L*** | ***+L*** | ***-L*** | ***+L*** |
| ***24h*** | ***17.29*** | ***15.22*** | ***13.88*** | ***11.25*** | ***10.50*** | ***8.52*** |
| ***48h*** | ***13.41*** | ***10.91*** | ***8.89*** | ***6.88*** | ***7.27*** | ***5.71*** |

**MOC2**

| ***IC50 (µg/ml)*** | ***Free Ce6*** | | ***mPCe6 Ms*** | | ***mPNCe6 Ms*** | |
| --- | --- | --- | --- | --- | --- | --- |
|  | ***- L*** | ***+ L*** | ***- L*** | ***+L*** | ***-L*** | ***+L*** |
| ***24h*** | ***18.46*** | ***15.41*** | ***13.99*** | ***10.73*** | ***11.24*** | ***8.89*** |
| ***48h*** | ***13.40*** | ***11.89*** | ***9.75*** | ***7.52*** | ***8.37*** | ***6.12*** |
